# Nanosized (Ni_1−*x*_Zn_*x*_)Fe_2_O_4_ for water oxidation†

**DOI:** 10.1039/c8na00200b

**Published:** 2018-10-22

**Authors:** Somayeh Mehrabani, Jitendra Pal Singh, Robabeh Bagheri, Abdul Ghafar Wattoo, Zhenlun Song, Keun Hwa Chae, Mohammad Mahdi Najafpour

**Affiliations:** Department of Chemistry, Institute for Advanced Studies in Basic Sciences (IASBS) Zanjan 45137-66731 Iran mmnajafpour@iasbs.ac.ir +98 24 3315 3201; Advanced Analysis Center, Korea Institute of Science and Technology (KIST) Seoul 02792 Republic of Korea; Key Laboratory of Marine Materials and Related Technologies, Zhejiang Key Laboratory of Marine Materials and Protective Technologies, Ningbo Institute of Materials Technology and Engineering, Chinese Academy of Sciences Ningbo 315201 China; Center of Climate Change and Global Warming, Institute for Advanced Studies in Basic Sciences (IASBS) Zanjan 45137-66731 Iran; Research Center for Basic Sciences & Modern Technologies (RBST), Institute for Advanced Studies in Basic Sciences (IASBS) Zanjan 45137-66731 Iran

## Abstract

Performing water splitting for H_2_ production is an interesting method to store different energies. For water splitting, an efficient and stable water-oxidizing catalyst is important. Ni–Fe (hydr)oxides are among the best catalysts for water oxidation in alkaline electrolytes. An Fe amount higher than 50% in Ni–Fe (hydr)oxides increases the overpotential for water oxidation. Thus, Ni–Fe (hydr)oxides with a high ratio of Fe to Ni have rarely been focused on for water oxidation. Herein, we report water oxidation using nanosized (Ni_1−*x*_Zn_*x*_)Fe_2_O_4_. The catalyst was characterized *via* some methods and tested at pH values of 3, 7 and 11 in phosphate buffer. Nanosized (Ni_1−*x*_Zn_*x*_)Fe_2_O_4_ is a good catalyst for water oxidation only under alkaline conditions. In the next step, amperometry studies showed current densities of 3.50 mA cm^−2^ and 11.50 mA cm^−2^ at 1.25 V in 0.10 M and 1.0 M KOH solution, respectively. The amperometric measurements indicated high catalyst stability in both 0.10 M and 1.0 M KOH. Tafel plots were obtained in KOH solution at concentrations of both 0.10 M and 1.0 M. At pH = 13 in KOH solution (0.10 M), linearity of lg(j) *vs.* potential was shown, with two slopes relating to both relatively low (170.9 mV per decade) and high overpotentials (484.2 mV per decade). In 1.0 M KOH solution, the Tafel plot showed linearity of lg(j) *vs.* potential, with two slopes relating to both relatively low (192.5 mV per decade) and high overpotentials (545.7 mV per decade). After water oxidation, no significant change was observed in the catalyst.

## Abstract 2

Nanosized (Ni_1−*x*_Zn_*x*_)Fe_2_O_4_ is a good catalyst for water oxidation under alkaline conditions.

## Graphical abstract

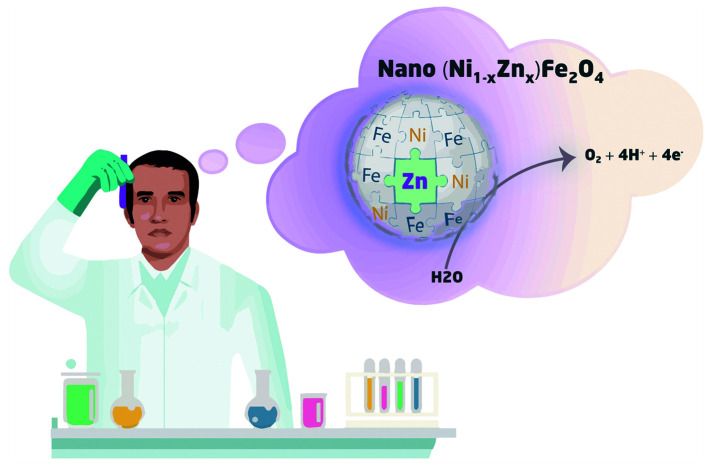

## Introduction

The post-oil economy requires that renewable and intermittent energy sources are efficiently and economically stored.^1^ The high-energy content of the H–H bond and environmental issues make H_2_ a promising fuel. Among different methods, water electrolysis for hydrogen production is very promising.^2–4^ In water electrolysis, the electrons for the reduction reaction are obtained by the water–oxidation reaction. On the other hand, water oxidation is one of the bottlenecks for water splitting. Thus, an efficient water-oxidizing catalyst is critical for water electrolyzers.^5–7^ Expensive metals have long been used as efficient catalysts for water electrolysis,^8^ but in large-scale production, the high cost is a problem. Thus, the search for alternative noble metal-free catalysts for the water–oxidation reaction is an active field.

For water electrolysis, current densities (j) higher than 1 mA cm^−2^ at low overpotentials are essential for water oxidation.^9^ Among the different Fe, Ni and Ni/Fe compounds,^10^ Ni/Fe (oxy)hydroxides are efficient catalysts for water oxidation in different electrolyte solutions.^11–30^ Such (oxy)hydroxides have the lowest overpotentials for water oxidation under alkaline conditions.^11–30^ In the 1980s, Corrigan's group studied the catalytic activities of Ni/Fe (oxy)hydroxides for water oxidation^11–13^ and synergic effects between Fe and Ni oxides toward water oxidation were reported in 1987.^11–13^ The role of Fe ions in Ni/Fe (oxy)hydroxides is an enigma. For Ni/Fe (oxy)hydroxides, the overpotential for water oxidation decreases when the amount of Fe increases from 0 to 10%, reaching a minimum in the range of 10% to 50%; for amounts of Fe higher than 50%, the overpotential further increases.^11^

Thus, nanoparticles with a high ratio of Fe to Ni have rarely been focused on for water oxidation.^11^ Herein, we report water oxidation using nanosized (Ni_1−*x*_Zn_*x*_)Fe_2_O_4_. In the structure, in addition to Fe and Ni as essential ions for water oxidation, the catalyst includes Zn ions as amphoteric ions that can be removed in the presence of KOH solution to provide more active sites on the surface of the catalyst.

## Results and discussion

The Pourbaix diagram of the system shows that:^30^

(i) At pH > 9, Ni(ii) oxide is the dominant form of Ni;^31^

(ii) Ni(ii) oxide at large positive biases is also stable;^31^

(iii) Fe(iii) ions are important species under oxidizing conditions.^31^

**Fig. 1:**
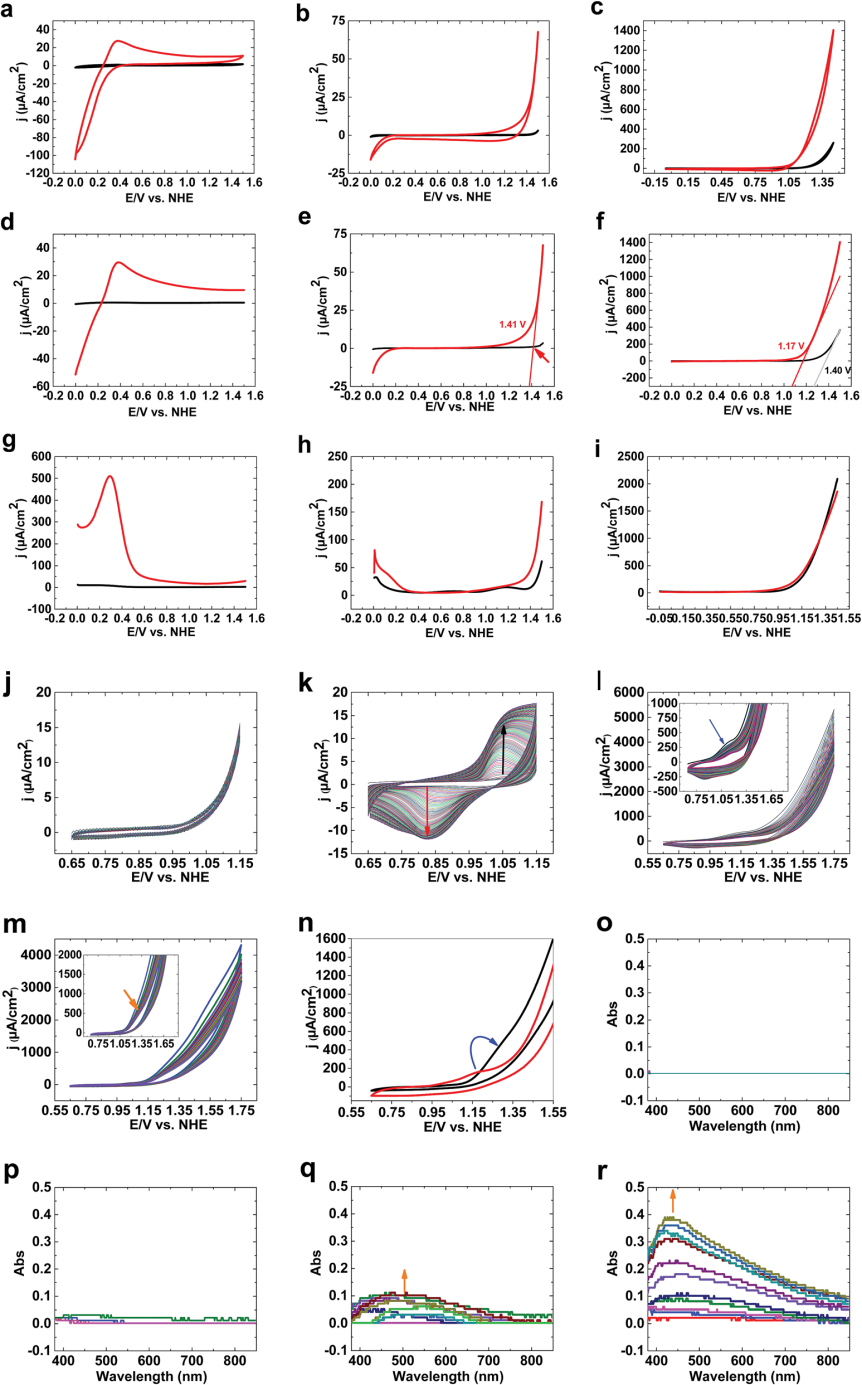
CVs of 1 (red) and bare FTO (black) at pH values of 3 (a), 7 (b) and 11 (c). LSVs of 1 (red) and bare FTO (black) at pH values of 3 (d), 7 (e) and 11 (f). SWVs of 1 (red) and bare FTO (black) at pH values of 3 (g), 7 (h) and 11 (i). Continuous CV scans of Fe(OH)_3_ on FTO in the absence (j) and presence (k) of Ni(ii) (pH 11). Continuous CV scans of Ni(OH)_2_ on FTO in the absence (l) and presence (m) of Fe(iii) (pH 11). CVs of Ni(OH)_2_ on FTO in the absence (red) and presence (black) of Fe(iii) (pH 11) (n). Spectroelectrochemistry data at pH 11 of bare FTO (o), and Fe(iii) saturated (p), Ni(ii) saturated (q) and Fe(iii)/Ni(ii) saturated (r) buffer phosphate solution. The experiments were carried out at room temperature with a conventional three-electrode setup, in which FTO, Ag|AgCl|KCl_sat_ (pH 3 and pH 7) or Hg|HgO (pH 11), and platinum foil served as the working, reference and auxiliary electrodes, respectively. The electrolyte for the electrochemical experiments was phosphate buffer (0.25 M; pH 3, 7 and 11), but for the spectroelectrochemistry measurements it was phosphate buffer (0.10 M; pH 11) to increase the concentration of Fe(iii) or Ni(ii) ions in solution.

CVs of 1 (see ESI†) under acidic conditions show no water-oxidizing activity compared to bare FTO (Fig. 1a). At pH 7, although bare FTO showed no activity for water oxidation (Fig. 1b), 1 showed low activity for water oxidation. Under these conditions, no oxidation peak was observed (Fig. 1b). At pH 11, high activity for 1 compared to bare FTO was observed (Fig. 1c). LSV studies of 1 at pH 3 showed no water oxidation (Fig. 1d). At pH 7, water oxidation was observed for 1; LSV studies showed an onset potential for water oxidation of 1.35 V (550 mV) (Fig. 1e).

The onset potential of water oxidation at pH 11 was 1.12 V (546 mV overpotential), which is promising for water oxidation (Fig. 1f). Square wave voltammetry studies displayed no clear peaks at pH 3, 7 and 11 at high potentials (>1) (Fig. 1g–i).

In the next step, we investigated the effects of Fe on the Ni oxide. At pH 11, Fe(OH)_3_ on FTO showed no clear oxidation/reduction peaks (Fig. 1j), although Fe(iii) oxidation to FeO_4_^2−^ could occur based on the Pourbaix diagram.^31^ Water oxidation by Fe oxyhydroxide films is low because of the poor electrical conductivity of thick films.^32*a*,*b*^ However, thin films with low iron loading exhibited high water-oxidizing activity.^32*c*^

After adding Ni(ii) to the buffer in the presence of Fe(OH)_3_ on FTO, Ni_3_(PO_4_)_2_ is precipitated, but a small amount of Ni(ii) is in solution. Under these conditions, and using Fe(OH)_3_ on FTO, a clear peak related to Ni(ii) to Ni(iii) oxidation was observed (Fig. 1k). In the next step, Ni(OH)_2_ on FTO was prepared, which showed a peak at 1.17 V related to the oxidation of Ni(ii)/(III) from a Ni(OH)_2_ structure (Fig. 1l). To investigate the effects of Fe(iii) on the electrochemistry of Ni(OH)_2_, we added a small amount of Fe(iii) ions to the buffer. The peak for the oxidation of Ni(ii) shifted to a higher potential (1.28 V), near to the onset of water oxidation (Fig. 1m and n). Such a shift, as displayed in Fig. 1n, significantly increases water oxidation. It suggests that Fe is suppressing Ni oxidation toward an oxidation potential closer to the onset potential of water oxidation, which has been also observed by Mukerjee,^33^ Dau and Strasser.^34^ We suggest that the shift in the oxidation of Ni(ii) to the water oxidation area could be important in increasing water oxidation because it could significantly couple Ni oxidation and water oxidation.

To investigate Ni oxidation, spectroelectrochemistry was used to detect Ni oxidation. The experiments indicated that although no change was observed for bare FTO (Fig. 1o) and Fe(iii) ions (Fig. 1p), Ni(ii) oxidation occurs in the absence and the presence of Fe(iii) at 1.15 V (Fig. 1q and r).

**Fig. 2:**
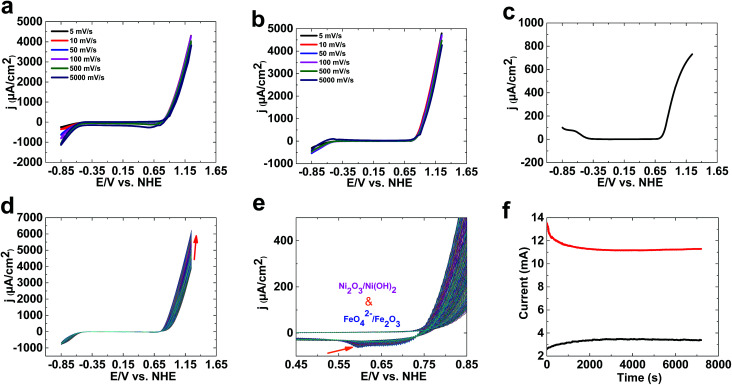
CVs of 1 at different scan rates at pH 13 (a). LSVs of 1 at different scan rates at pH 13 (b). A SWV of 1 at pH 13 (c). Continuous CV scans of 1 at a scan rate of 100 mV s^−1^ at pH 13 (d and e). Amperometry data for 1 at 1.25 V at pH 13 (black) and 14 (red) (f). The experiments were carried out at room temperature with a conventional three-electrode setup, in which FTO, Hg|HgO and platinum foil served as the working, reference and auxiliary electrodes, respectively. The electrolyte for the electrochemical experiments was KOH solution (0.10 M or 1.0 M).

As shown in Fig. 1, 1 is only a good catalyst for water oxidation under alkaline conditions. In the next step, we tested the activity of 1 in KOH (0.10 M). High activity was observed for 1 under these conditions. LSV, SWV, and CV studies indicated no oxidation peak before water oxidation (Fig. 2a–c). An increase in LSV and CV scan rates has no significant effect on water oxidation. The onset potential of water oxidation at pH 13 was 0.90 V (overpotential: 448 mV), which is moderate for water oxidation (Fig. 2a). The overpotential for the onset of water oxidation in KOH (1.0 M) is 400 mV.

Continuous CV scans show that the catalytic activity of 1 increases from 3890 to 6213 μA cm^−2^ at 1.25 V (Fig. 2d and e). A reduction peak at 0.45 V was observed, which based on Pourbaix diagrams^31,32^ could be related to FeO_4_^2−^/Fe_2_O_3_ and Ni(OH)_3_/Ni(OH)_2_ reduction. The broad peak indicates that both types of ion could be reduced under these conditions.

Amperometry showed current densities of 3500 μA cm^−2^ and 11 500 μA cm^−2^ at 1.3 V for 0.10 M and 1.0 M KOH, respectively (Fig. 2f). The amperometric measurements indicated the high stability of 1 in both 0.10 M and 1.0 M KOH.

**Fig. 3:**
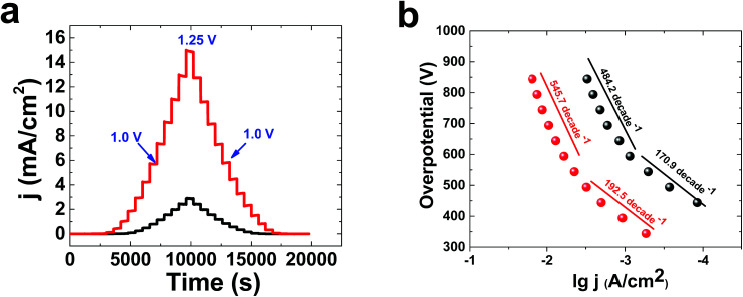
The multistep amperometry data for 1 at pH 13 (black) and 14 (red) (a). A comparison of Tafel plots of 1 at pH 13 (black) and 14 (red) (b). The experiments were carried out at room temperature with a conventional three-electrode setup, in which FTO, Hg|HgO and platinum foil served as the working, reference and auxiliary electrodes, respectively. The electrolyte for the electrochemical experiments was KOH solution (0.10 M or 1.0 M).

Multistep amperometry^35^ is an interesting method to find the current at different potentials (Fig. 3a). lg j (A cm^−2^)/overpotential, or Tafel, plots were obtained for 1 in KOH solution at concentrations of both 0.1 M and 1.0 M (Fig. 3b). The Tafel behaviour of 1 in KOH solution (0.10 M) showed linearity of lg(j) *vs.* potential, with two slopes related to both relatively low (170.9 mV per decade) and high (484.2 mV per decade) overpotentials. In KOH solution (1.0 M), the Tafel plot of 1 displayed linearity of lg(j) *vs.* potential, with two slopes related to both relatively low (192.5 mV per decade) and high (545.7 mV per decade) overpotentials. The high number of bubbles on the surface of the electrode at high potentials in KOH is important to the high value of the Tafel slope. For molecular catalysts in solution, a slope of about 59 mV per decade is usually observed. The Tafel slope depends on electron transport, mass transport and gas bubbles. Under these conditions, it is suggested that Nafion could limit electron and mass transport.

**Fig. 4:**
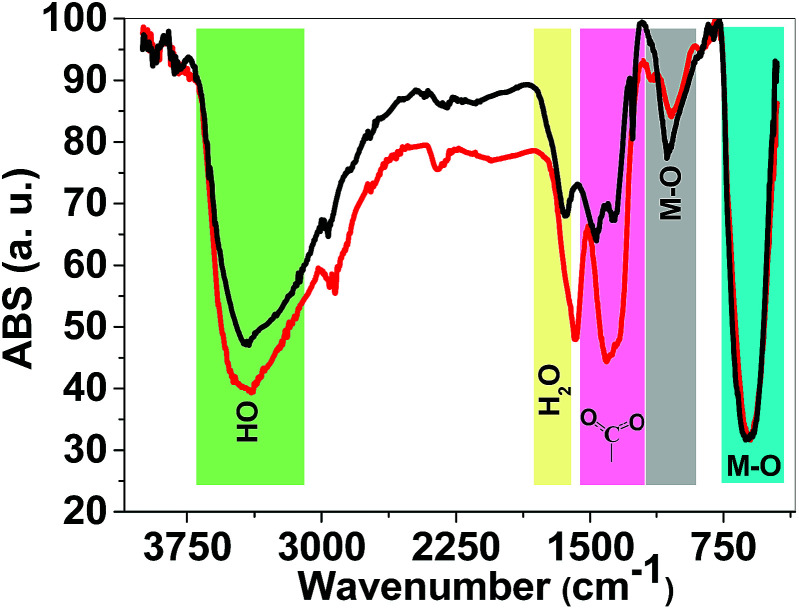
FTIR spectra of 1 (red) and 2 (black). FTIR spectra were obtained using KBr pellets.

FTIR spectroscopy is a reliable method to identify M–O bonds in metal oxides.^36^ FTIR spectra of both 1 and 2 are shown in Fig. 4 and show stretching vibrations of sharp M–O octahedra at 617 and 1060 cm^−1^. The peaks at 1300–1450 cm^−1^ are related to carboxylate groups from precursors. FTIR spectra also showed broad peaks at ∼3000–3600 cm^−1^ attributed to antisymmetric and symmetric O–H stretching modes. The FTIR spectra of 1 and 2 are very similar and no significant changes were observed.

**Fig. 5:**
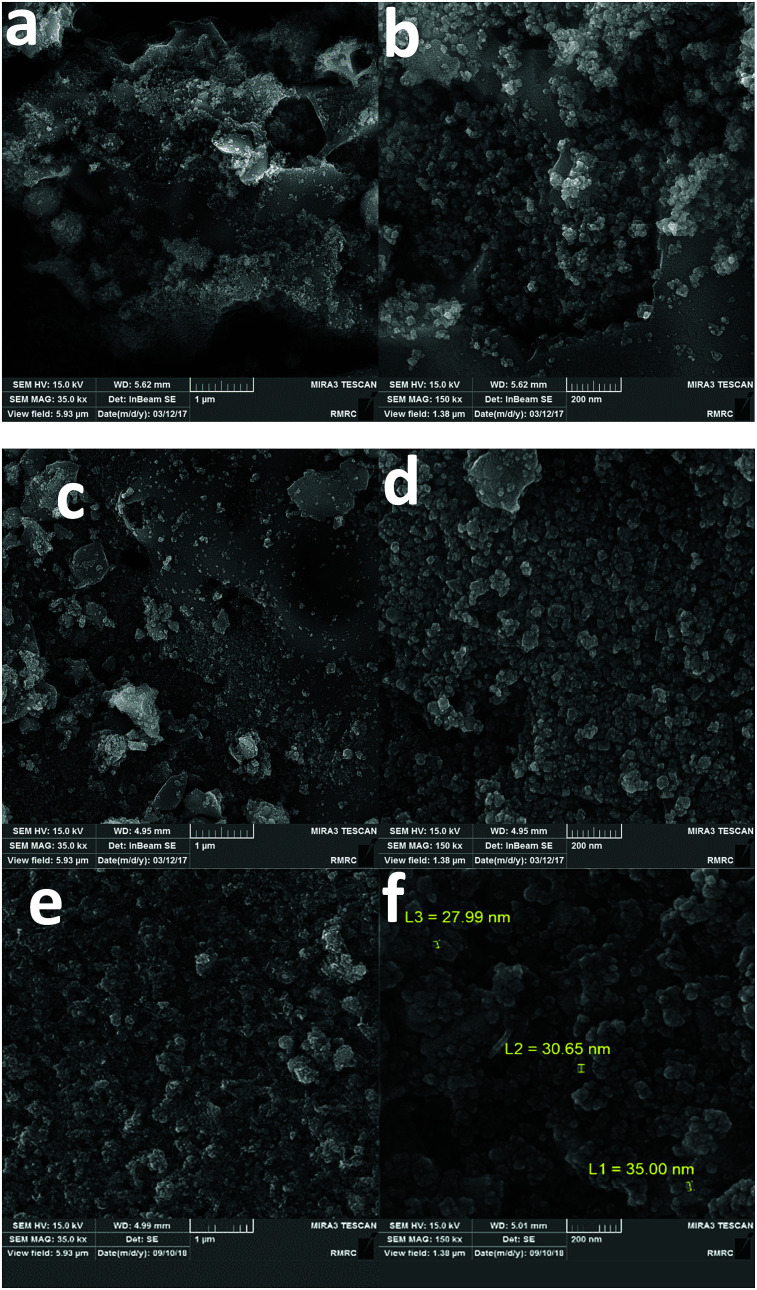
SEM images of 1 (a and b) and 2 (c and d). SEM images of the nanoparticles obtained after water oxidation at 1.25 V for 72 hours in the presence of KOH (1.0 M) (e and f).

Scanning electron microscopy (SEM) images of both 1 and 2 indicated nanosized particles (10–100 nm). For 2, more clear boundaries between nanoparticles could be observed (Fig. 5, S1 and S2, ESI†). The obtained nanoparticles after water oxidation at 1.25 V for 72 hours in the presence of KOH (1.0 M) showed nanosize (20–50 nm) (Fig. 5 and S3, ESI†), and Zn, Fe and Ni elements (Fig. S6, ESI†).

**Fig. 6:**
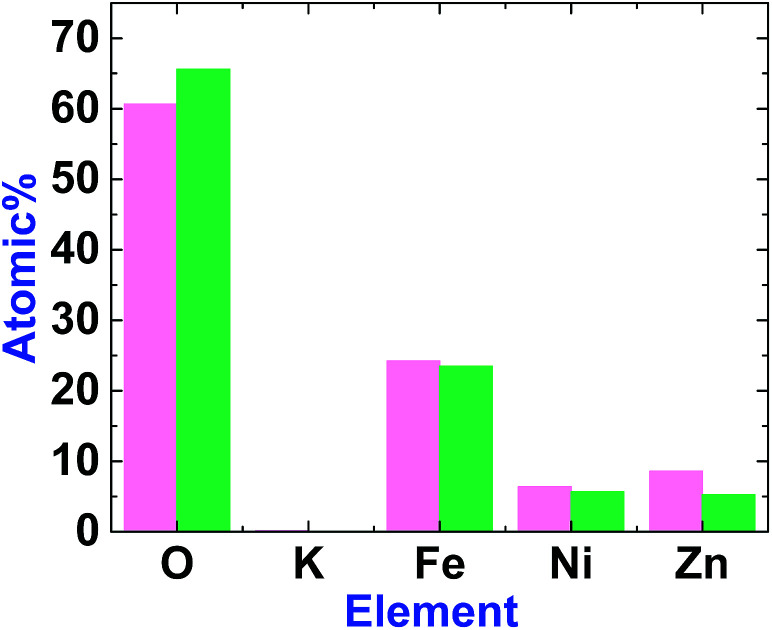
The graph shows the element amounts in 1 (magenta) and 2 (green). The amount of Zn decreases after treatment.

EDX-SEM studies showed Fe, Ni and Zn ions on the surfaces of 1 and 2. However, the amount of Zn in 2 is less than in 1 (Fig. 6, S3 and S4, ESI†). This could be attributed to the amphoteric character of Zn(ii), which is soluble in both acidic and alkaline solutions.

**Fig. 7:**
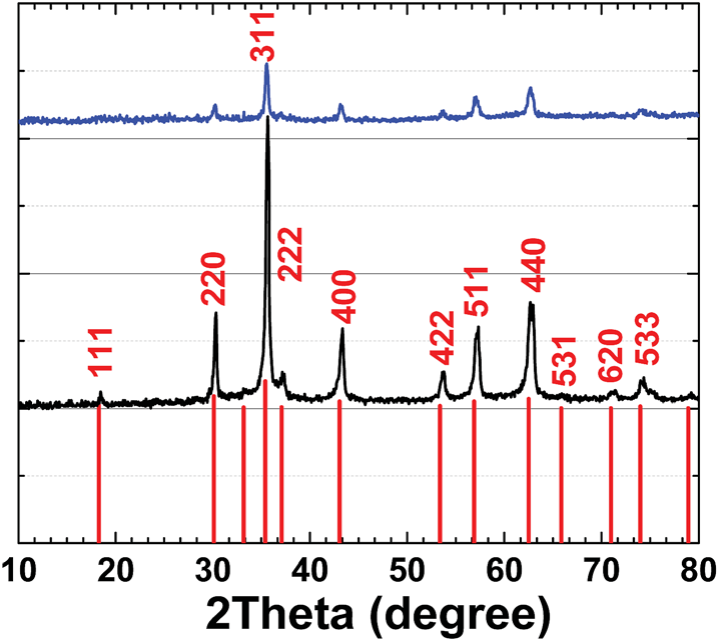
XRD patterns of (Ni_1−*x*_Zn_*x*_)Fe_2_O_4_ (ref.: 00-008-0234) (red), 1 (black) and 2 (blue).

XRD patterns for 1 and 2 are very similar and are related to (Ni_1−*x*_Zn_*x*_)Fe_2_O_4_ (ref.: 00-008-0234) (Fig. 7).

Using the Scherrer equation:^37^crystallite size (average) = *Kλ*/(*B* cos *Θ*)where *B*: structural broadening; *K*: shape factor; *Θ*: Bragg angle; and *λ*: the X-ray wavelength, a size of 150–200 nm was calculated for both 1 and 2. The XRD pattern of 2 showed no significant change compared to 1.

**Fig. 8:**
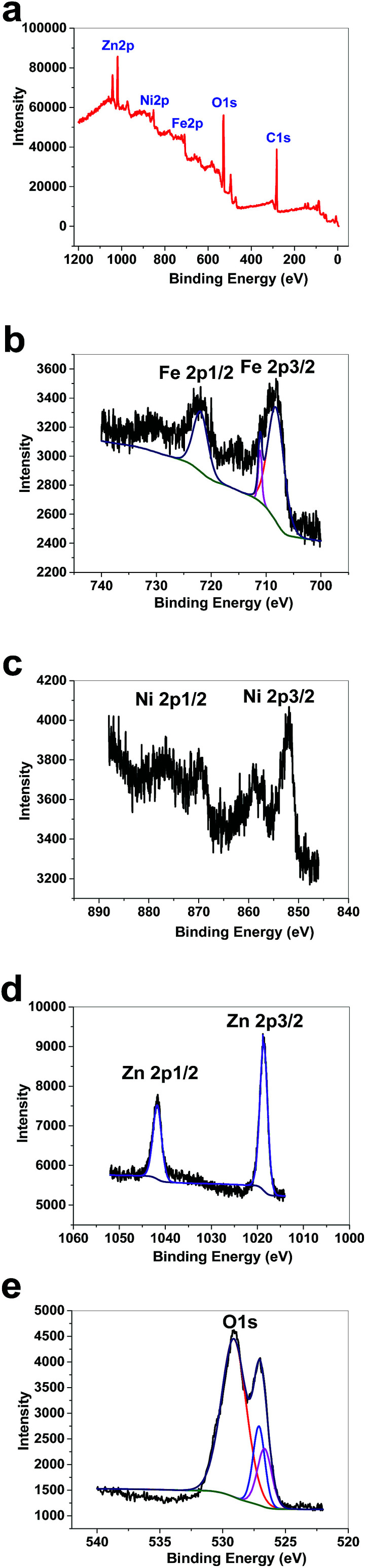
XPS data for 1 (a). XPS data in the Fe area for 1 (b). XPS data in the Ni area for 1 (c). XPS data in the Zn area for 1 (d). XPS data in the O area for 1 (e).

XPS data for 1 showed a large contribution from oxygen on the surface of the compound. Zn, Fe and Ni were also detected *via* the technique (Fig. 8a). The peaks in the Fe 2p region have a split spin–orbit component (13.0 eV) and are observed at ∼708.5 and 710.9 eV (Fe 2p_3/2_) and 721 eV (Fe 2p_1/2_); these are related to both Fe(ii) and Fe(iii) (Fig. 8b).^38^ The peaks in the Ni 2p region have a significant split spin–orbit component (17.8 eV). In 1, Ni 2p_3/2_ showed a peak at 852.11 eV, which is attributed to Ni^2+^ (Fig. 8c).^39^ In 1, Ni 2p_1/2_ showed a weak peak at 869.95 eV (Fig. 8c). Satellites could be observed in the Ni area. The peaks in the Zn 2p region have a split spin–orbit component (23.0 eV) and are observed at ∼1018.63 (Zn 2p_3/2_) and 1041.63 eV (Zn 2p_1/2_); these are related to Zn(ii) (Fig. 8d).^40^ The peak at ∼1018 eV indicates a significant change in the chemical environment compared to ZnO (1021.80 eV) (Fig. 8d). It was proposed that the binding of Zn(ii) to Fe(iii)–O results in the shifting of the binding energy.^41^ The area attributed to O 1s showed different peaks attributed to OH_2_, OH and O on the surface of 1 (Fig. 8e).

**Fig. 9:**
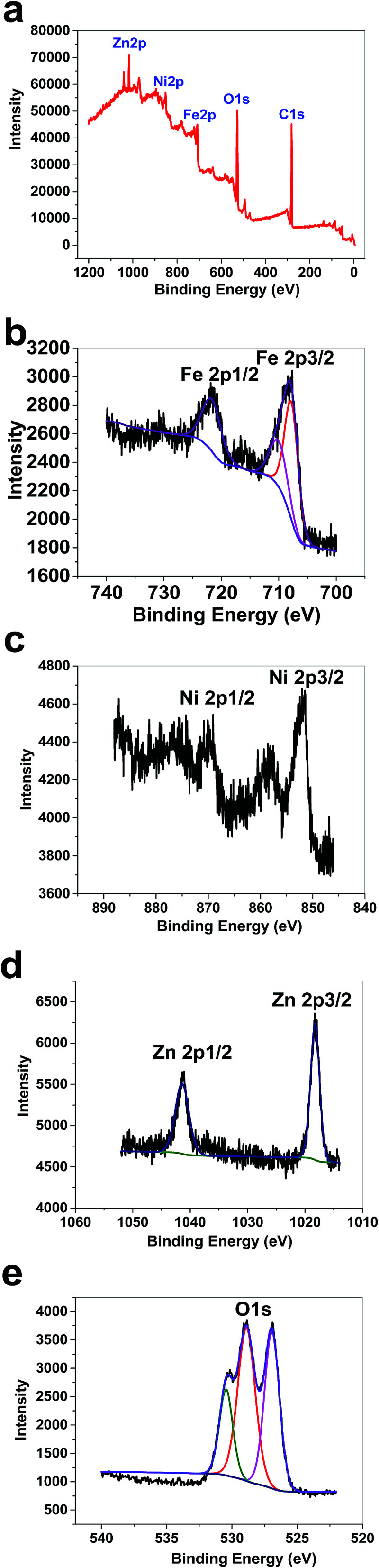
XPS data for 2 (a). XPS data in the Fe area for 2 (b). XPS data in the Ni area for 2 (c). XPS data in the Zn area for 2 (d). XPS data in the O area for 2 (e).

XPS data for 2 displayed a large contribution from oxygen on the surface of the compound. Zn, Fe and Ni ions were also detected using XPS (Fig. 9a). The peaks in the Fe 2p region have a split spin–orbit component (13.0 eV) and are observed at ∼708.0 and 710.43 eV (Fe 2p_3/2_) and 722 eV (Fe 2p_1/2_); these are related to both Fe(ii) and Fe(iii) (Fig. 9b). The peaks in the Ni 2p region have a split spin–orbit component (17.8 eV). In 2, Ni 2p_3/2_ showed a peak at 851.62 eV and another at 853.38 eV, which are attributed to Ni^2+^ (Fig. 9c).^22^ In 2, Ni 2p_1/2_ showed a weak peak at 870 eV (Fig. 9c). Satellites could be observed in the Ni area. The peaks in the Zn 2p region have a split spin–orbit component (23.0 eV) and are observed at ∼1018.63 (Zn 2p_3/2_) and 1041.63 eV (Zn 2p_1/2_); these are related to Zn(ii) (Fig. 9d). The area attributed to O 1s showed different peaks attributed to OH_2_, OH, and O on the surface of 2 (Fig. 9e).

**Fig. 10:**
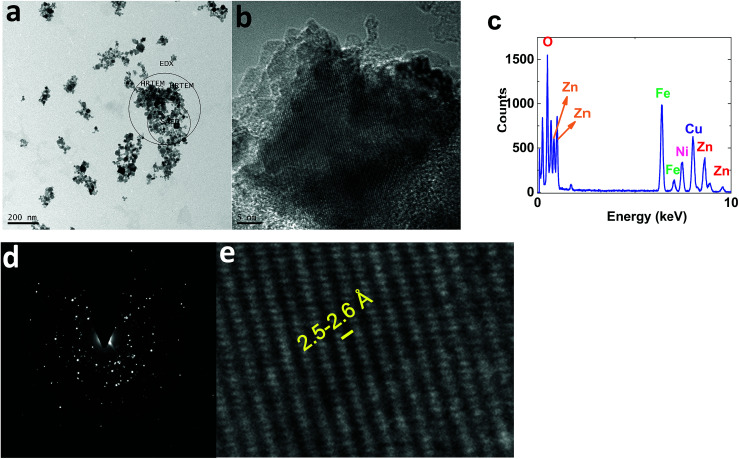
TEM (a) and HRTEM (b and e) images, the EDX-TEM spectrum (c), and the SAED pattern (d) of 1.

TEM images of 1 displayed nanoparticles (10–50 nm) (Fig. 10a). HRTEM images showed interplanar spacing of 2.5–2.6 Å, attributed to (Ni_1−*x*_Zn_*x*_)Fe_2_O_4_ (ref.: 00-008-0234; 2*Θ* = 35.7°) (Fig. 10b and e).

EDX-TEM studies showed Zn, Fe and Ni elements in the nanoparticles (Fig. 10c).

The selected area (electron) diffraction (SAED) pattern^42^ of 1 consists of rings, centered on a bright central spot, which indicates undiffracted electrons, and is related to the pattern obtained from a polycrystalline material (Fig. 10d). Each ring corresponds to planes of different orientation and different interplanar spacing, attributed to (Ni_1−*x*_Zn_*x*_)Fe_2_O_4_ (ref.: 00-008-0234) (Fig. 10d).

**Fig. 11:**
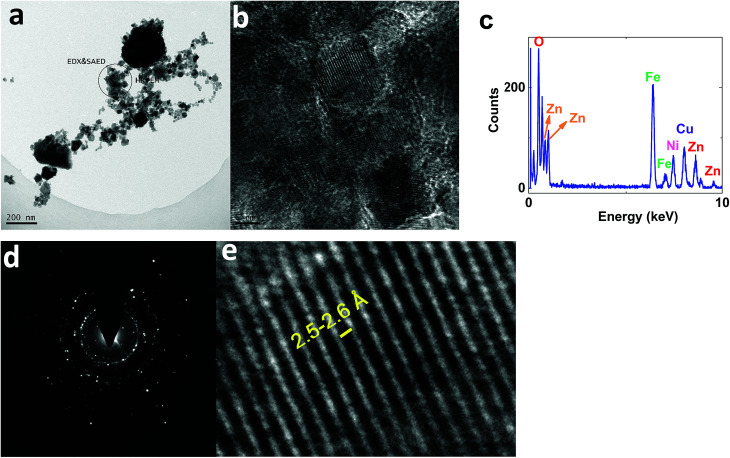
TEM (a) and HRTEM (b and e) images, the EDX-TEM spectrum (c), and the SAED pattern (d) of 2.

On the other hand, TEM images of 2 showed nanoparticles (10–50 nm) (Fig. 11a). HRTEM indicated interplanar spacing of 2.5–2.6 Å, attributed to (Ni_1−*x*_Zn_*x*_)Fe_2_O_4_ (ref.: 00-008-0234; 2*Θ* = 35.7°) (Fig. 11b and e). However, amorphization was observed around the crystallized structure. Such amorphization was also observed *via* XRD. EDX-TEM studies showed Zn, Fe and Ni elements in the nanoparticles (Fig. 11c).

SAED studies, shown in Fig. 11d, showed a pattern similar to 1, attributed to (Ni_1−*x*_Zn_*x*_)Fe_2_O_4_ (ref.: 00-008-0234).

**Fig. 12:**
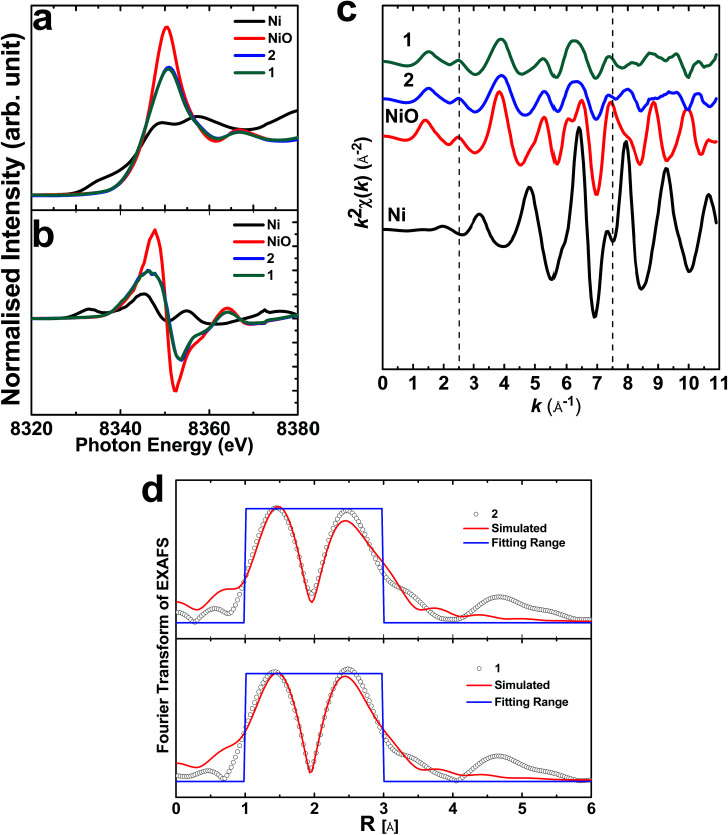
XANES (a) and the first derivative (b) spectra of the materials. The *k*-weight EXAFS spectra of the materials (c) and the simulated Fourier transform EXAFS spectra (d).

Further, to throw light on the local electronic and atomic structures of 1 and 2, X-ray absorption near edge structure (XANES) measurements of the Ni K-edge were performed for these materials. The results from these measurements are shown in Fig. 12. XANES (Fig. 12a) and its first derivative counterpart (Fig. 12b) reveal that Ni exists in the same valence state as the coincidence of the main edge of the Ni K-edge spectra. *k*-Weight EXAFS spectra for these materials have no similarity either with NiO or Ni, which reveals that Ni ions exist in a different coordination to NiO or Ni (Fig. 12c). The spectra of these materials seem to analogues of each other. Simulated Fourier transforms of the EXAFS spectra of these materials are shown in Fig. 12d. The simulation parameters are shown in Table 1. It is clear that the Ni^2+^ ions are surrounded by six O^2−^ ions, representing the octahedral sites of the spinel structures of both 1 and 2.

**Table 1:** Coordination numbers and radial distances of Ni–O and Ni–Ni bonds in 1 and 2

Material		Coordination number				
		*N*	*R*	*σ* ^2^	*Є*_o_ (eV)	*R*-Factor
2	Ni–O	7.1 ± 2.3	2.04	0.013 ± 0.013	−1.05	0.037
	Ni–Ni	7.1 ± 2.3	2.99 ± 0.03	0.006 ± 0.007	−1.05	
1	Ni–O	7.2 ± 2.9	2.01	0.015 ± 0.009	−3.6	0.043
	Ni–Ni	7.2 ± 2.9	2.98 ± 0.02	0.007 ± 0.005	−3.6	

Nitrogen adsorption–desorption isotherms were used to study both 1 and 2 (Fig. S7–S16, ESI†). A nitrogen adsorption–desorption isotherm is a plot of relative pressure *vs.* volume adsorbed, measured from the amount of nitrogen gas that adsorbs onto the surface of a compound, and the subsequent amount that desorbs at a constant temperature (Fig. S15a†).^43^ The nitrogen adsorption–desorption isotherm of 1 is typical for a mesoporous material. The Brunauer–Emmett–Teller (BET) method is used to find the surface area for a model of adsorption that incorporates a multilayer coverage model. A BET plot resulted in a value for the total specific surface area of 28.8 m^2^ g^−1^, a mean pore diameter of 31.7 nm and a total pore volume of 0.23 cm^3^ g^−1^ (Fig. S15b†). The Barrett–Joyner–Halenda (BJH) method is used for calculating pore size distributions from experimental isotherms, and applies to mesopore and small macropore size ranges (Fig. S15c†). From the BJH plot, it can be observed that 1 has mesopores with a radius of 1.2 to 100 nm, and has distribution peaks at 1.2, 3.6 and 26 nm. The integrated pore volume (*V*_p_) is 0.2246 cm^3^ g^−1^. The pore size distribution, based on the DH-plot method, is also shown in Fig. S15d.† As shown in Fig. S15d,† it can be concluded that 1 has mesopores with a radius of 1.6 to 100 nm, with distribution peaks at 2.1, 10.6 and 39.1 nm. The integrated pore volume (*V*_p_) for 1 is 0.2367 cm^3^ g^−1^ and the integrated pore area (*A*_p_) is 20.8 m^2^ g^−1^.

The nitrogen adsorption–desorption isotherm of 2 is also typical for a mesoporous material (Fig. S16a†). The application of the BET model resulted in a value for the total specific surface area of 26.8 m^2^ g^−1^, a mean pore diameter of 13.1 nm and a total pore volume of 0.09 cm^3^ g^−1^ (Fig. S16b†). The BJH method data for 2 is shown in Fig. S16c.†

The BJH plot showed that 2 has a mesoporous structure with a radius of 1.2 to 19.2 nm, and has distribution peaks at 1.2 and 7.1 nm. The integrated pore volume (*V*_p_) is 0.084 cm^3^ g^−1^.

A pore size distribution based on the DH-plot method is also shown in Fig. S16d.† As shown in Fig. S16d,†2 has mesopores with a radius of 1.2 to 16.4 nm, with distribution peaks at 1.2 and 6.0 nm. The integrated pore volume (*V*_p_) for 2 is 0.089 cm^3^ g^−1^ and the integrated pore area (*A*_p_) is 24.5 m^2^ g^−1^.

Comparing both sets of data indicates a few changes after conversion, which occur because of water oxidation and (or) the effects of KOH on the surface of the catalyst. The small pore diameter of 2 compared to 1 could be related to the placement of ions from the electrolyte on the surface of the catalysts. However, as we observed, the changes have no effect on the water-oxidizing activity of the catalyst.

## Conclusions

Of the metal oxides, Ni/Fe (oxy)hydroxides are promising and efficient catalysts for water oxidation in alkaline electrolyte solutions. Nanosized nickel/zinc/iron oxide, (Ni_1−*x*_Zn_*x*_)Fe_2_O_4_, was placed on FTO with Nafion and used for water oxidation. At a minimum of pH 11, the spectroelectrochemistry data indicated that Ni(ii) oxidation occurs in the absence and presence of Fe(iii) at 1.15 V. We observed a shift in the oxidation of Ni(ii) toward the water oxidation area in the presence of Fe(iii) ions and suggest that this shift could be important to increase water oxidation because it could significantly couple Ni oxidation and water oxidation.

These nanosized particles were characterized *via* FTIR, SEM, XRD, (HR)TEM and XPS studies before and after water oxidation, and the experiments showed that there were no significant changes after water oxidation. Current densities of 3.5 mA cm^−2^ and 11.5 mA cm^−2^ at 1.25 V in 0.10 M and 1.0 M KOH, respectively, were observed for the catalyst. High stabilities for the catalyst in both 0.10 M and 1.0 M KOH were observed. Tafel plots at pH = 14 in KOH solution (1.0 M) indicated the linearity of lg(j) *vs.* potential, with two slopes related to both relatively low (192.5 mV per decade) and high (545.7 mV per decade) overpotentials.

## Conflicts of interest

There are no conflicts to declare.

## Supplementary Material

NA-001-C8NA00200B-s001
