# Supplementary material for: Nanosized (Ni1−xZnx)Fe2O4 for water oxidation
Source: Nanoscale Adv. 2018 Oct 22;1(2):686–95. doi: 10.1039/c8na00200b (PMC9473301; doi:10.1039/c8na00200b)
Supplement: NA-001-C8NA00200B-s001 [file NA-001-C8NA00200B-s001.pdf]

Electronic Supplementary Material (ESI) for Nanoscale Advances.
This journal is © The Royal Society of Chemistry 2018

Electronic Supplementary Information

Nanosized $(Ni_{1-x}Zn_x)Fe_2O_4$ for water oxidation

Somayeh Mehrabani,[a] Jitendra Pal Singh,[b] Robabeh Bagheri,[c] Abdul Ghafar Wattoo,[c] Zhenlun Song[c], Keun Hwa Chae[b] and Mohammad Mahdi Najafpour*[a,d,e]

[a]Department of Chemistry, Institute for Advanced Studies in Basic Sciences (IASBS), Zanjan, 45137-66731, Iran

[b]Advanced Analysis Center, Korea Institute of Science and Technology (KIST), Seoul 02792, Republic of Korea

[c]Surface Protection Research Group, Surface Department, Ningbo Institute of Materials Technology and Engineering, Chinese Academy of Sciences, 519 Zhuangshi Road, Ningbo 315201, China

[d]Center of Climate Change and Global Warming, Institute for Advanced Studies in Basic Sciences (IASBS), Zanjan, 45137-66731, Iran

[e]Research Center for Basic Sciences & Modern Technologies (RBST), Institute for Advanced Studies in Basic Sciences (IASBS), Zanjan 45137-66731, Iran

*Corresponding author; Phone: (+98) 24 3315 3201; E-mail: mmnajafpour@iasbs.ac.ir

Experimental

Materials

All reagents and solvents were purchased from the commercial sources and used without further purifications. Nickel zinc iron oxide, $(Ni_{1-x}Zn_x)Fe_2O_4$, (nanopowder, <100 nm particle size (BET), ≥99% trace metals basis) was purchased from Sigma-Aldrich Company.

Synthesis

1: Nickel/zinc/iron oxide, $(Ni_{1-x}Zn_x)Fe_2O_4$, (nanopowder, <100 nm particle size (BET), ≥99% trace metals basis) was purchased from Sigma-Aldrich Company.

2: water oxidation at 1.25 V was performed for **1** after 24 hours in the presence of KOH (1.0 M). Then, the particles were washed with water.

Characterization

Electrochemical experiments were performed using an $EmStat^{3+}$ from PalmSens (Netherlands). Cyclic voltammetry studies were carried out with a conventional three-electrode setup, in which FTO, $Ag|AgCl|KCl_{sat}$ and a platinum foil served as working, reference and auxiliary electrodes, respectively. The distance between two opposite sides of the FTO electrode were measured by a digital caliper MarCal 16ER model (Mahr, Germany).

20 µL of **1** or **2** was dispersed in water (20.0 mg/mL) and dripped on the FTO electerode (1.0 cm^2) and dried at 70 °C. Then, 10 µL of 0.5 wt % Nafion solution was cast on the surface of the FTO electrode (1.0 cm^2).

The temperature was measured by Laserliner 082 (Germany). X-ray photoelectron spectroscopy (XPS) measurements were done on an X-ray BesTec XPS system (Germany) with an AlK_α X-ray source (hυ =1486.6 eV). SEM was carried out with a LEO 1430VP. For

TEM, the samples were studied using a Philips CM120. X-ray powder diffraction patterns were recorded with a Bruker D8 ADVANCE (Germany) diffractometer (CuK_{α} radiation).

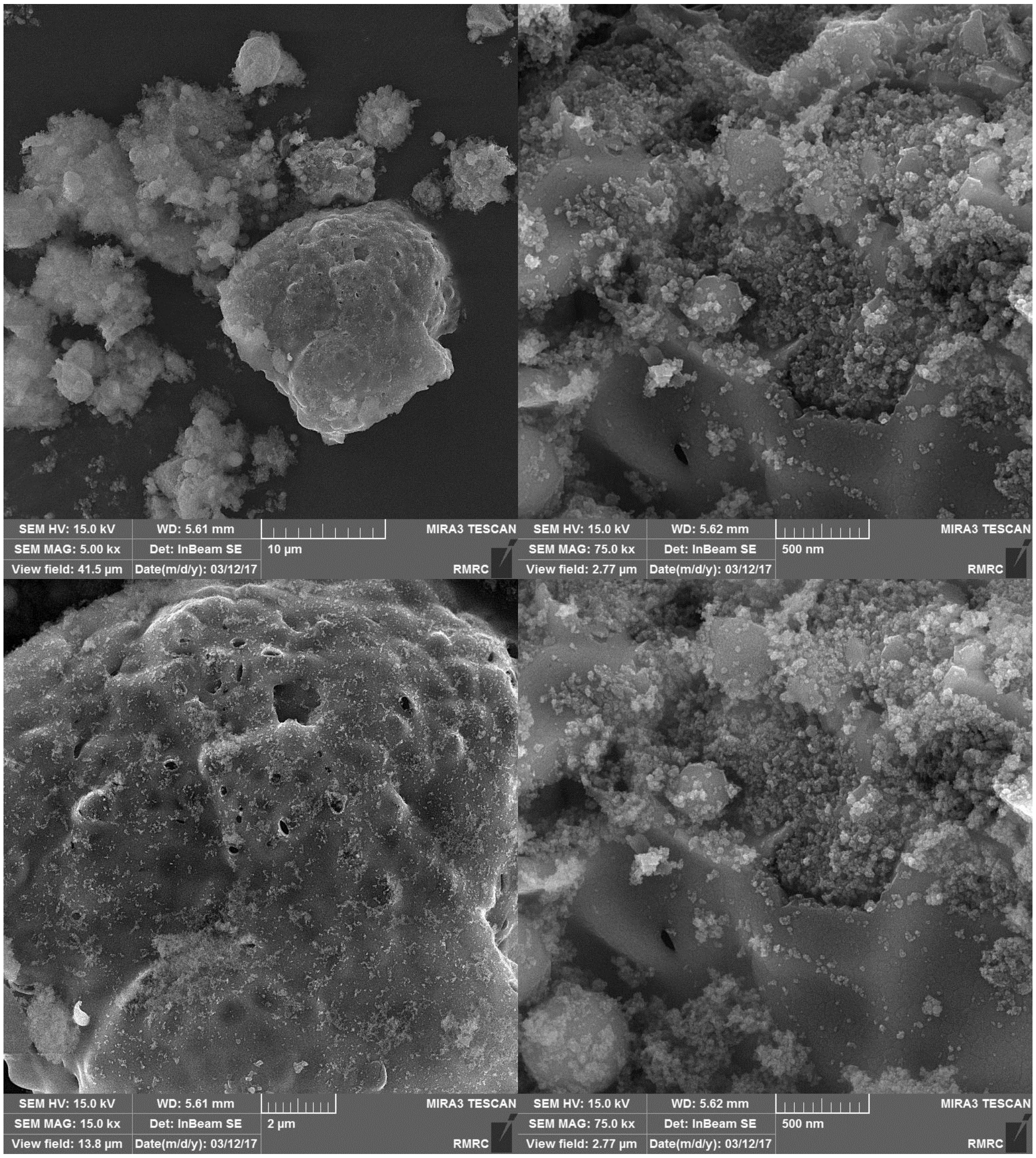

Figure S1 SEM images of **1**.

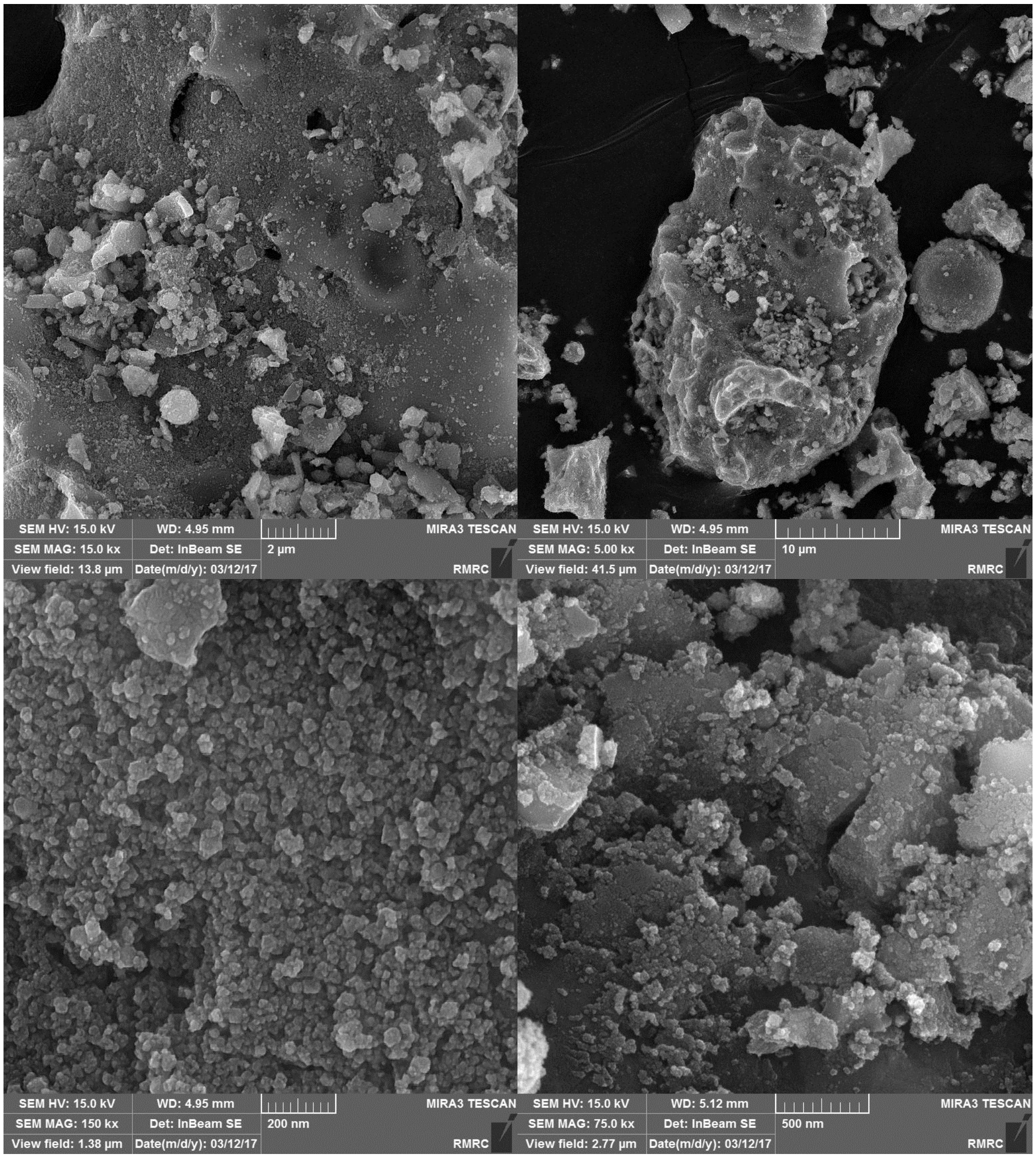

Figure S2 SEM images of **2**.

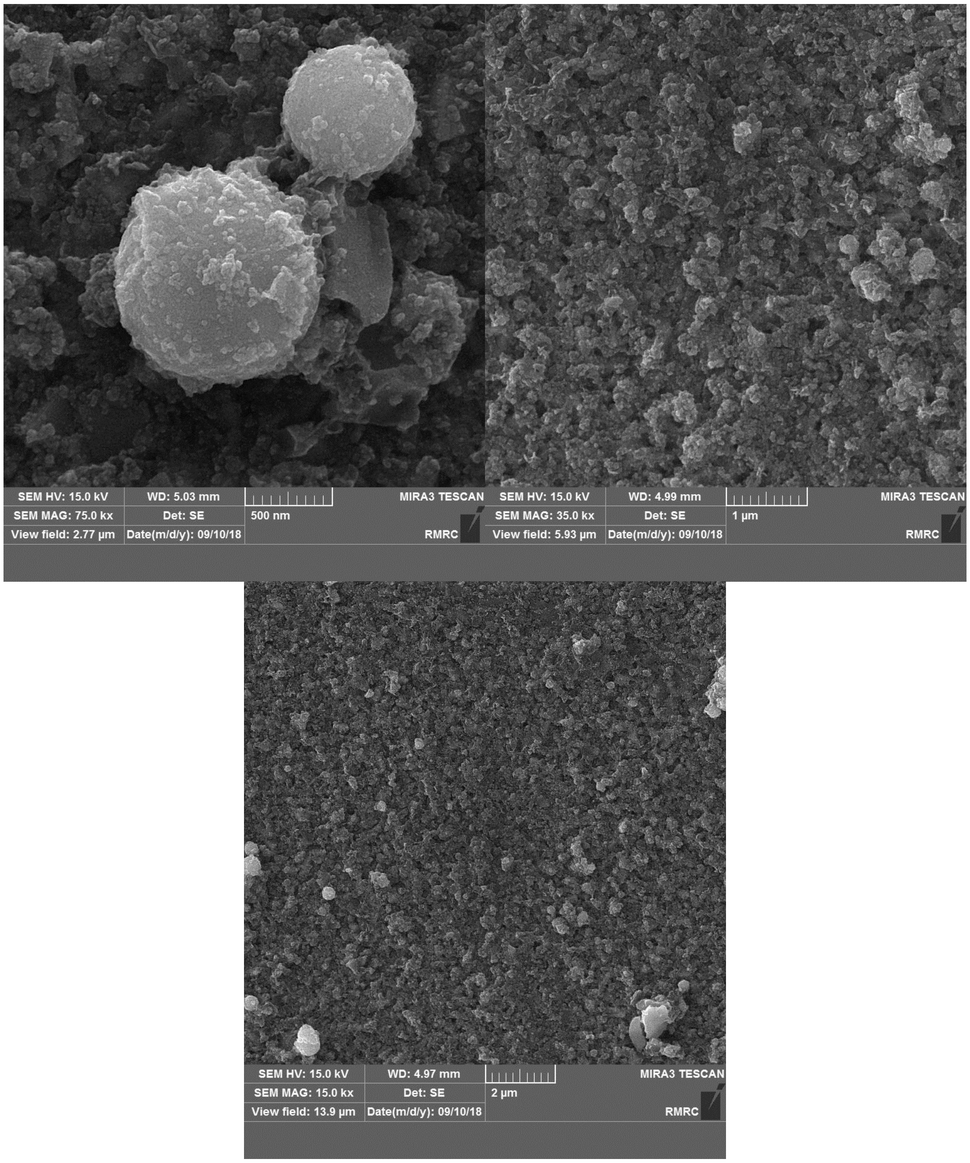

Figure S3 SEM image of the obtained nanoparticles after water oxidation at 1.25 V after 72 hours in the presence of KOH (1.0 M).

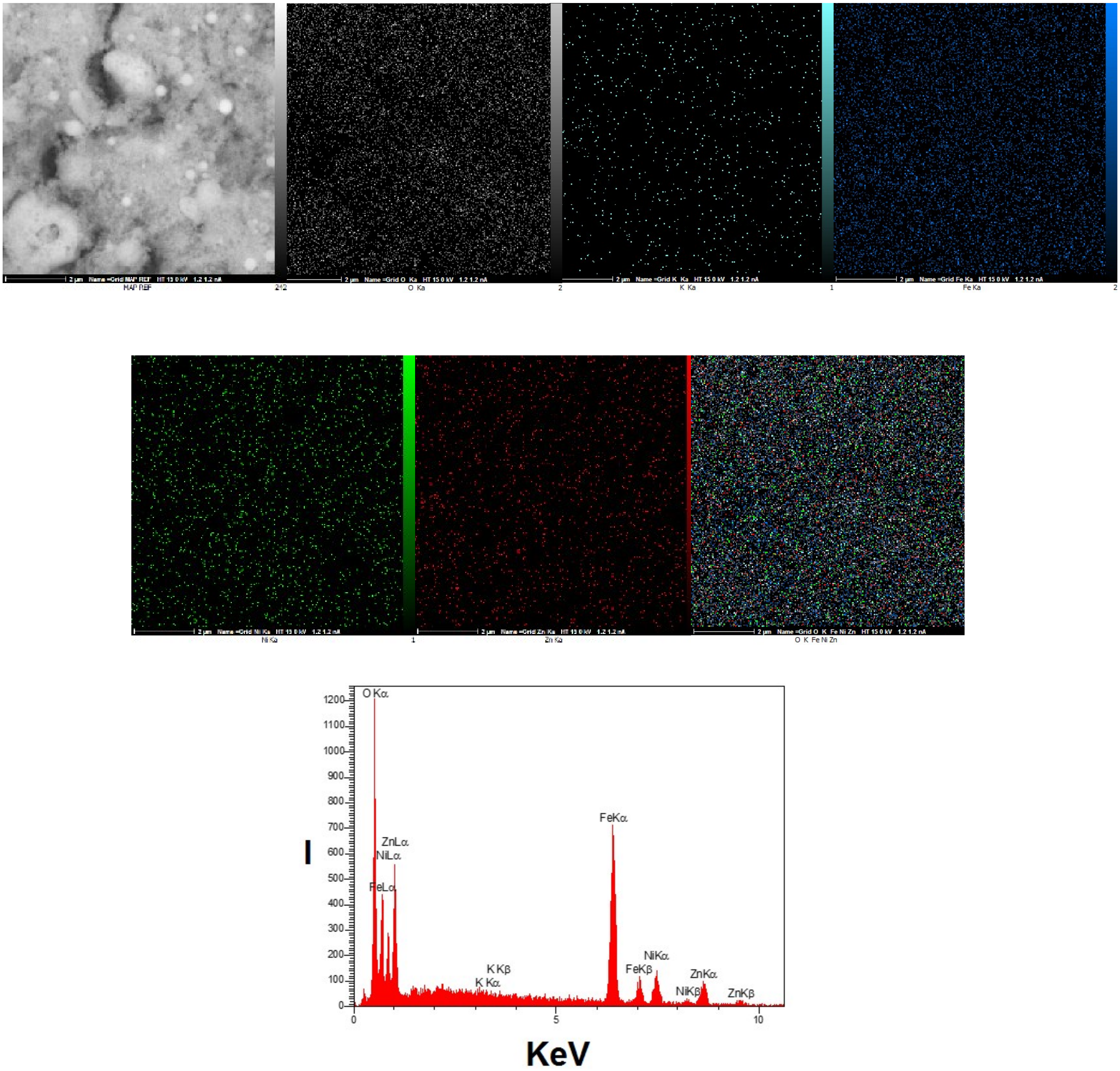

Figure S4 EDX-Mapping and spectrum of **1**.

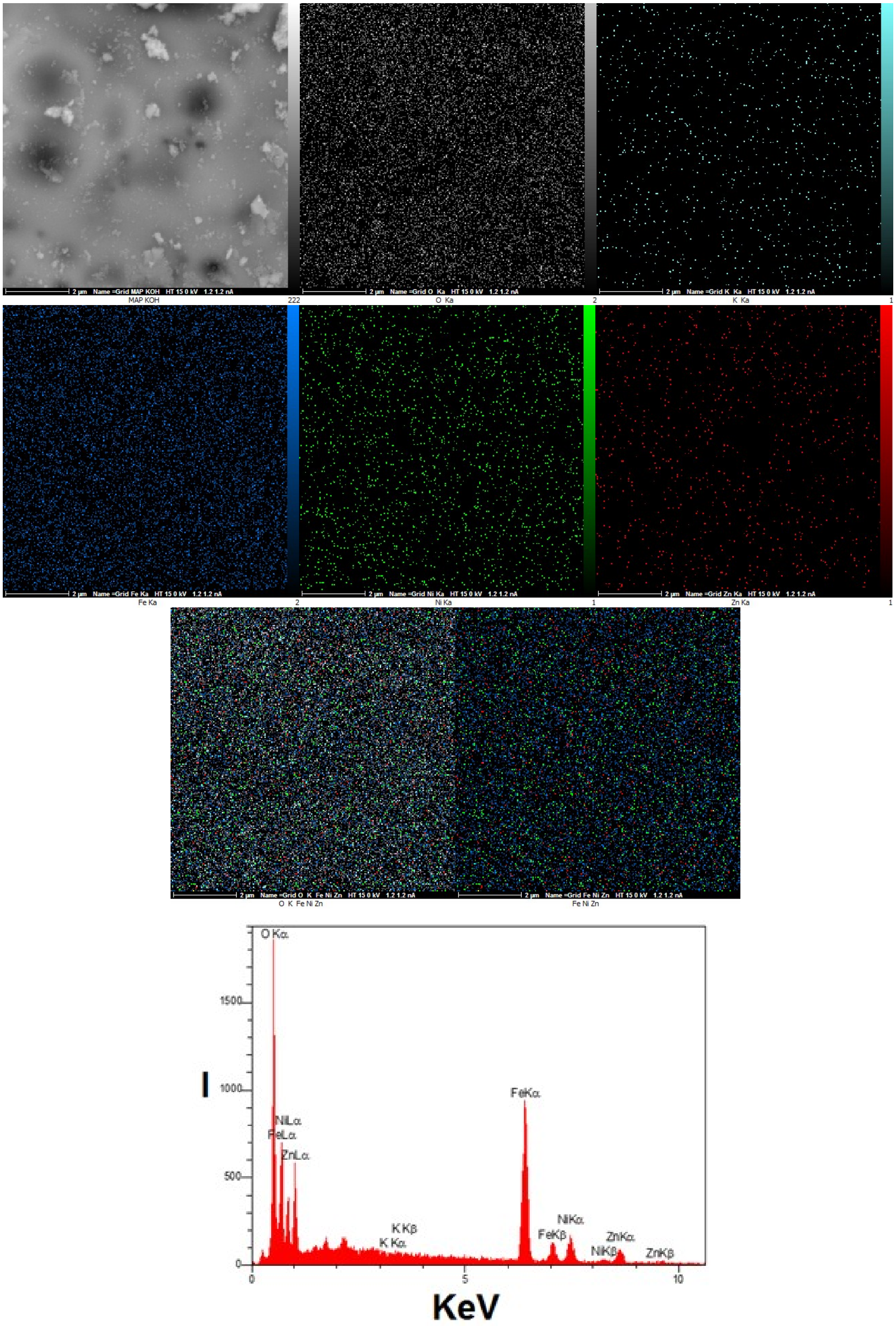

Figure S5 EDX-Mapping and spectrum of **2**.

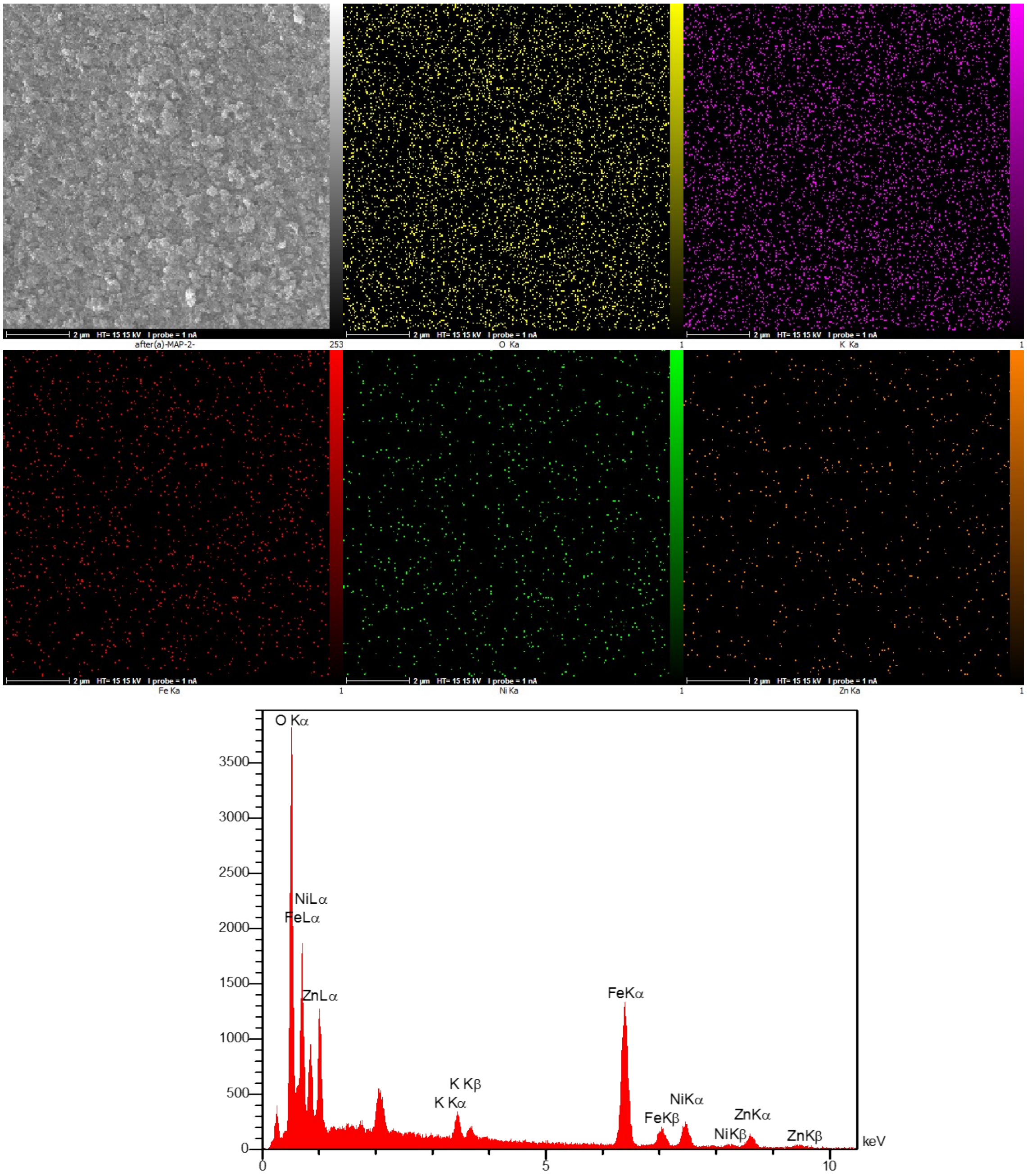

Figure S6 EDX-Mapping image of the obtained nanoparticles after water oxidation at 1.25 V after 72 hours in the presence of KOH (1.0 M).

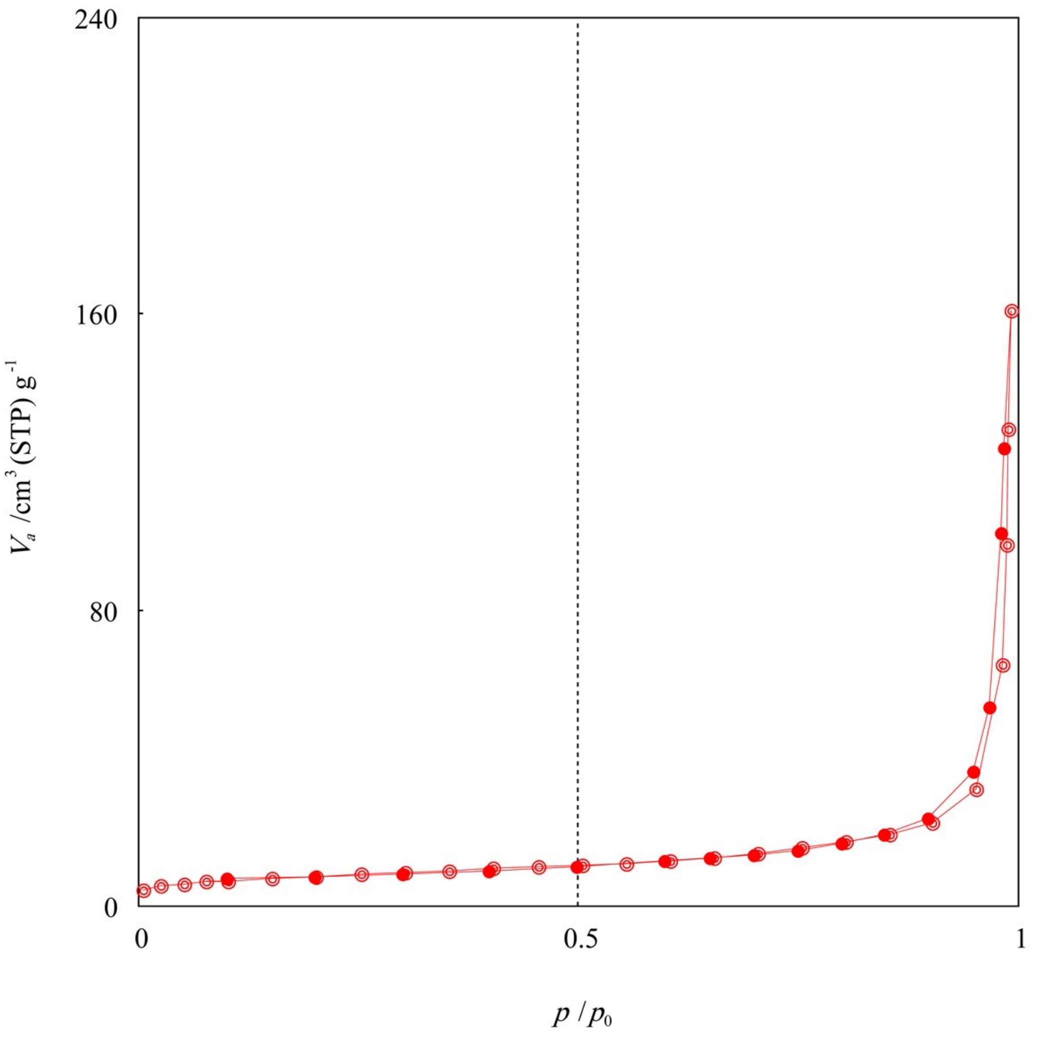

Adsorption / desorption isotherm

Adsorptive N2

Adsorption temperature 77 [K]

S.M.Ref.DAT

S.M.Ref
maryam akbari
pretreatment-5h-100degree

Sample weight	2.1900E-02	[g]	Date of measurement	17/04/24
Saturated vapor pressure	87.674	[kPa]	Time of measurement	9:35:47

Figure S7 The nitrogen adsorption-desorption isotherms for **1**.

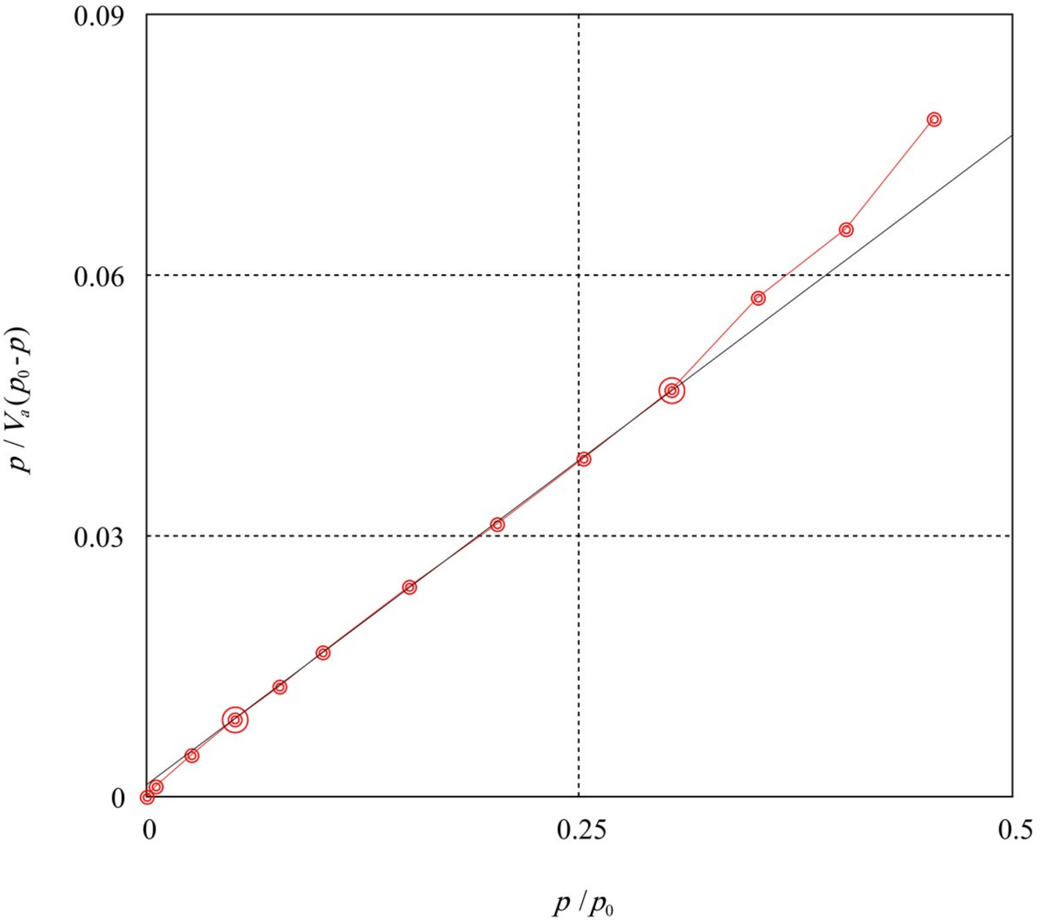

BET-Plot

Adsorptive N2

Adsorption temperature 77 [K]

◎ S.M.Ref.DAT

S.M.Ref
maryam akbari
pretreatment-5h-100degree

Sample weight	2.1900E-02	[g]	Date of measurement	17/04/24
Saturated vapor pressure	87.674	[kPa]	Time of measurement	9:35:47

V_m	6.6206	[cm^3(STP) g^{-1}]	$a_{s,BET}$	2.8816E+01	[m^2 g^{-1}]
C	116.43		Total pore volume(p/p_0=0.990)	0.2284	[cm^3 g^{-1}]
Mean pore diameter	31.700	[nm]			

Figure S8 The Brunauer, Emmett, and Teller (BET) plot for **1**.

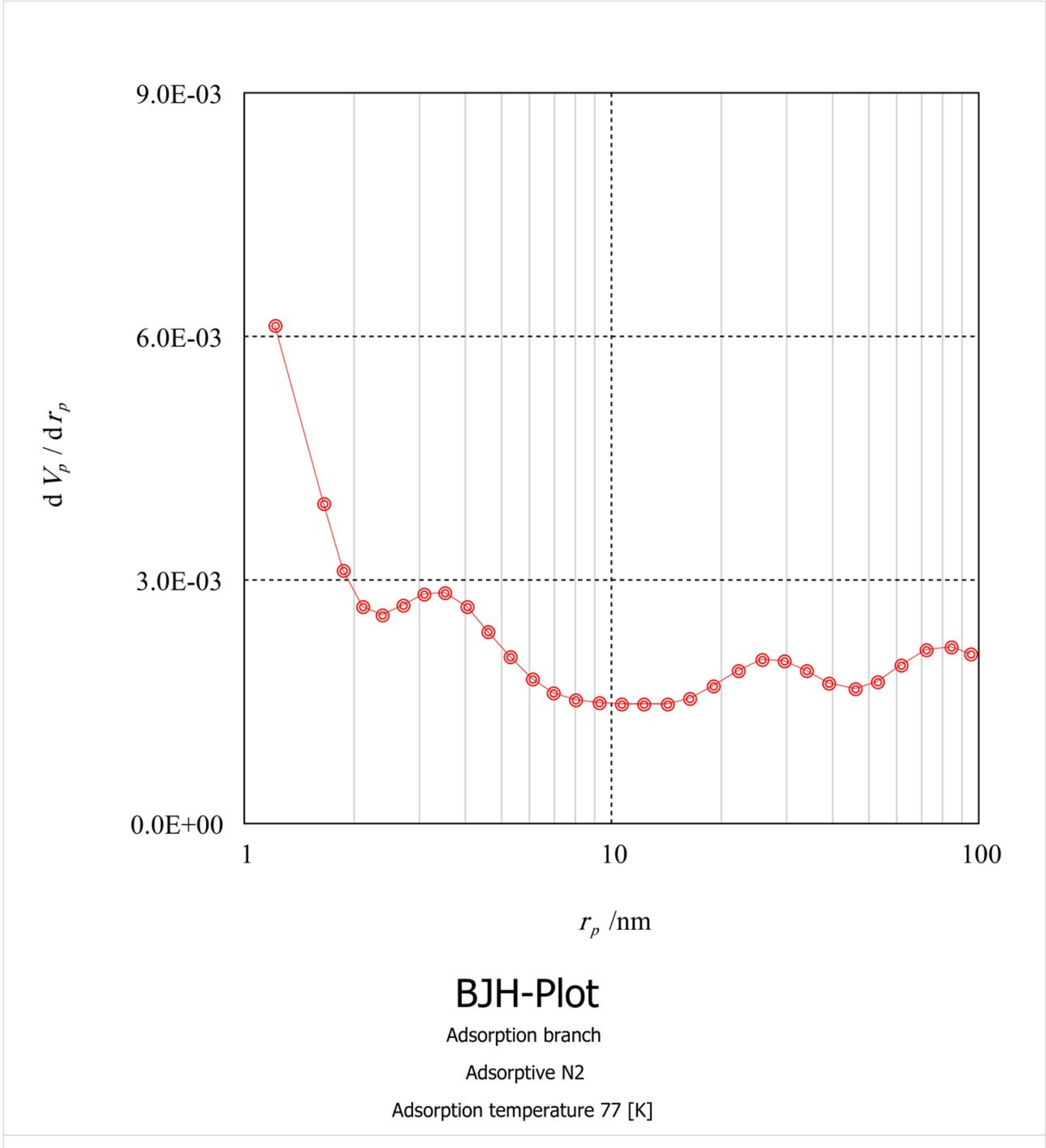

Figure S9 The Barrett, Joyner, and Halenda (BJH) plot for **1**.

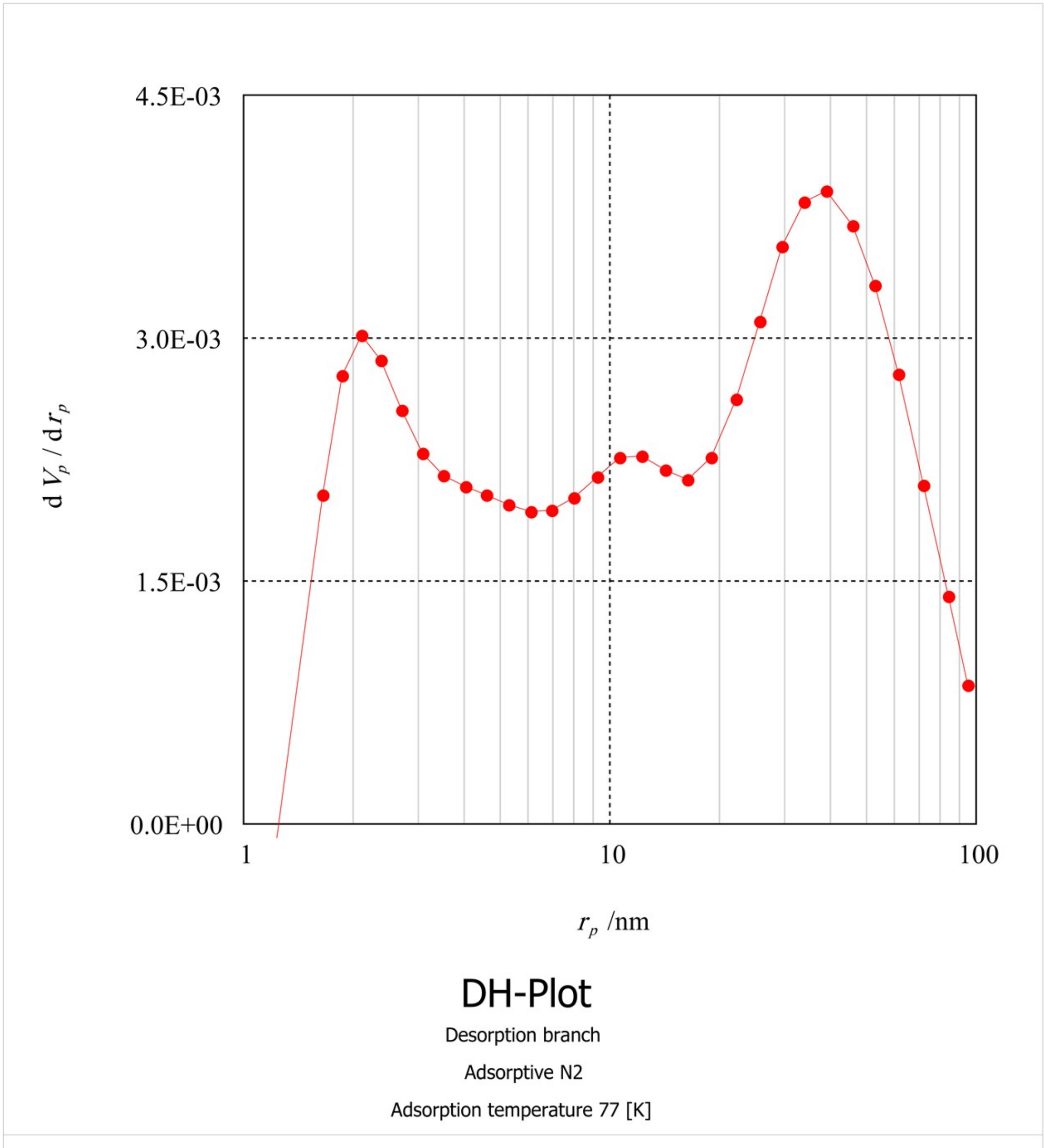

Figure S10 The DH plot for **1**.

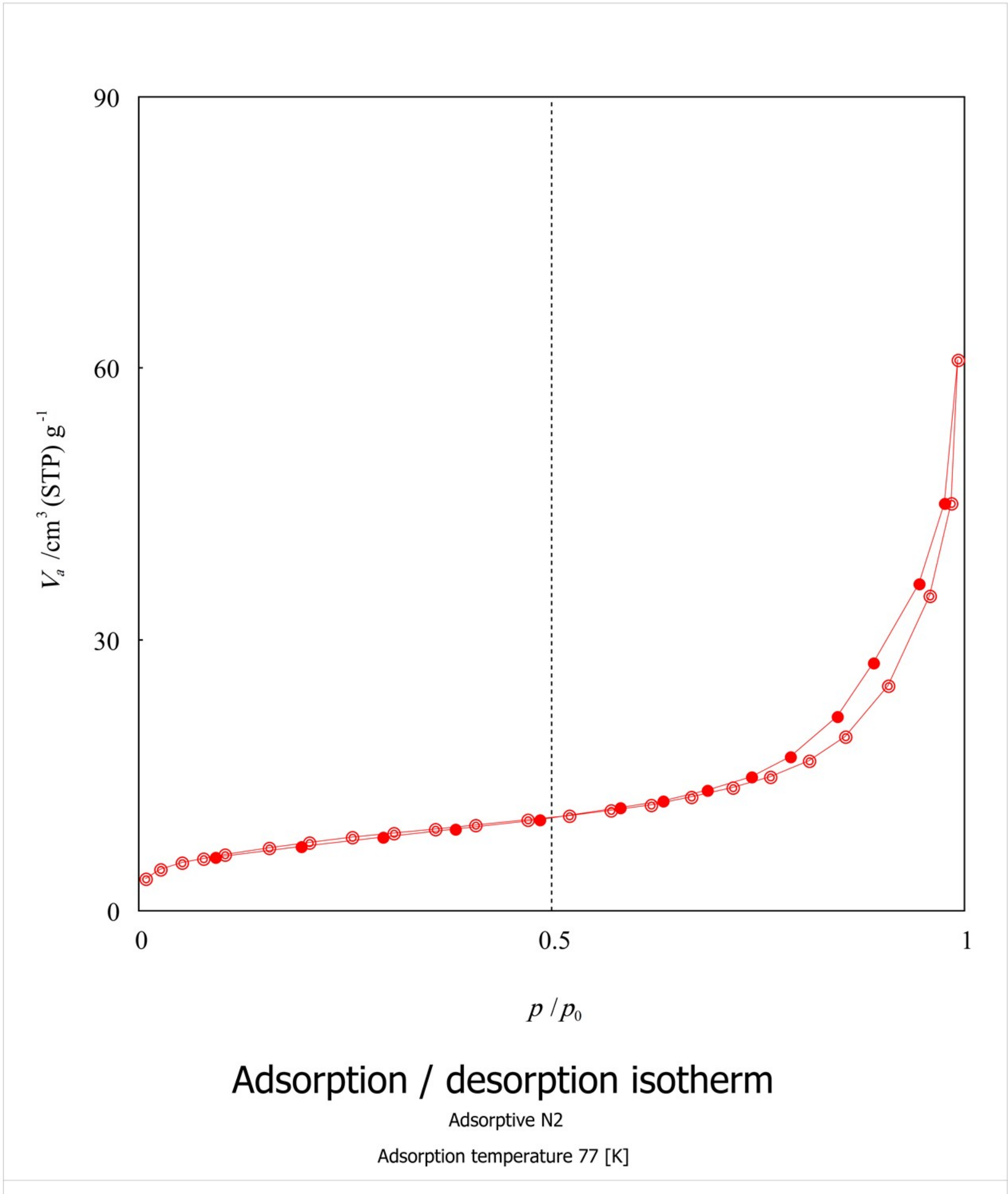

Figure S11 The nitrogen adsorption-desorption isotherms for **2**.

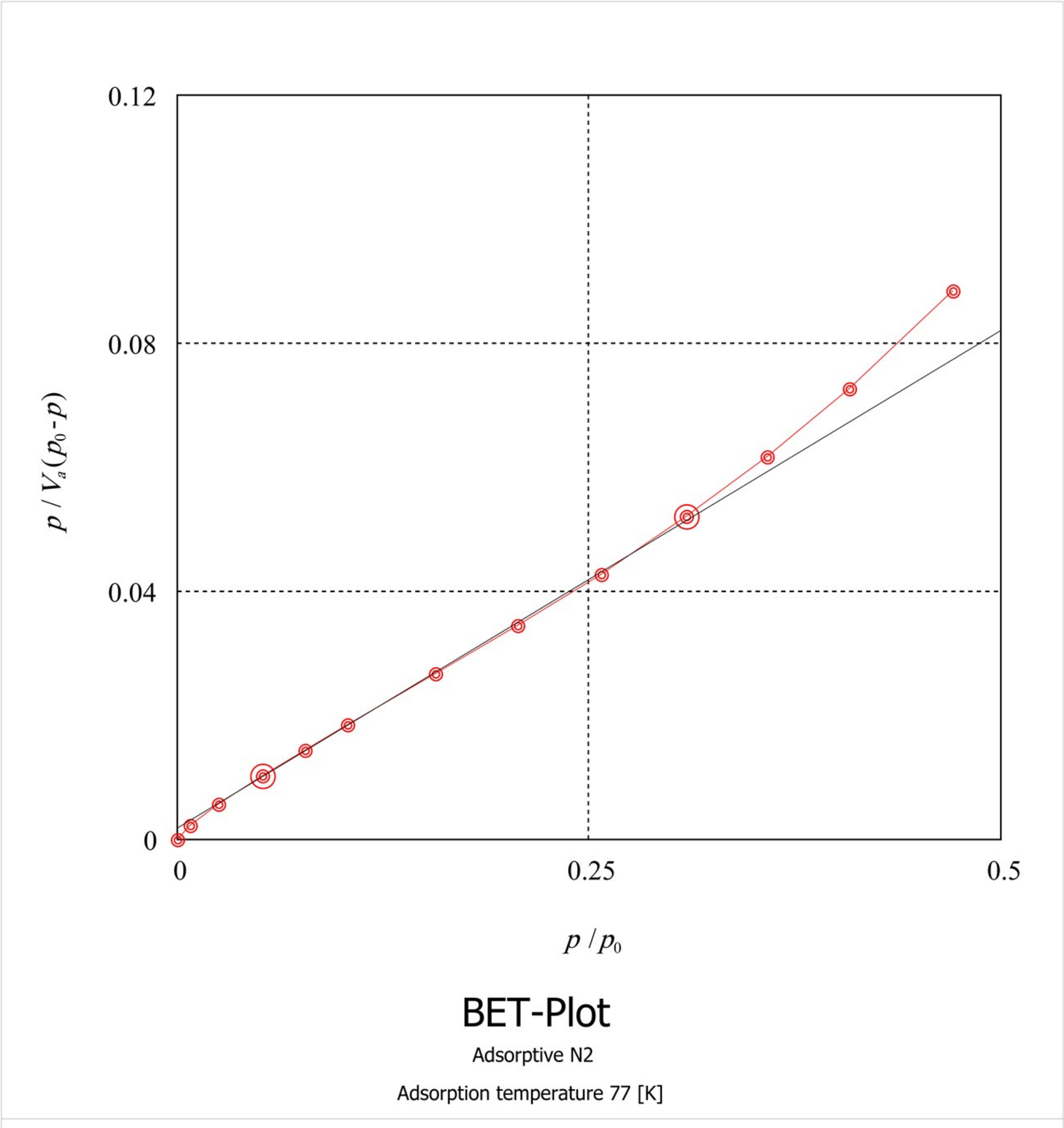

Figure S12 The Brunauer, Emmett, and Teller (BET) plot for **2**.

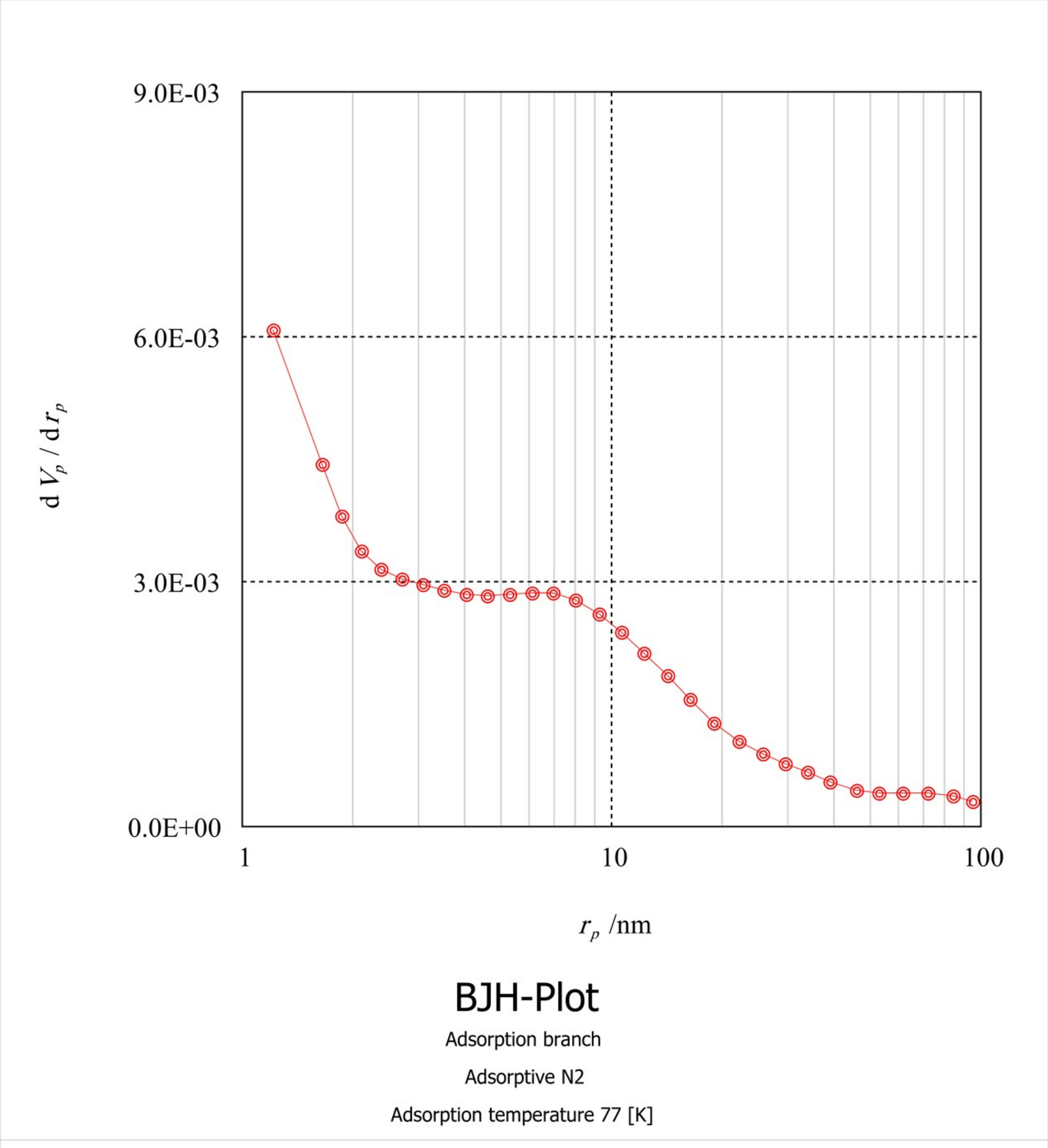

Figure S13 The Barrett, Joyner, and Halenda (BJH) plot for **2**.

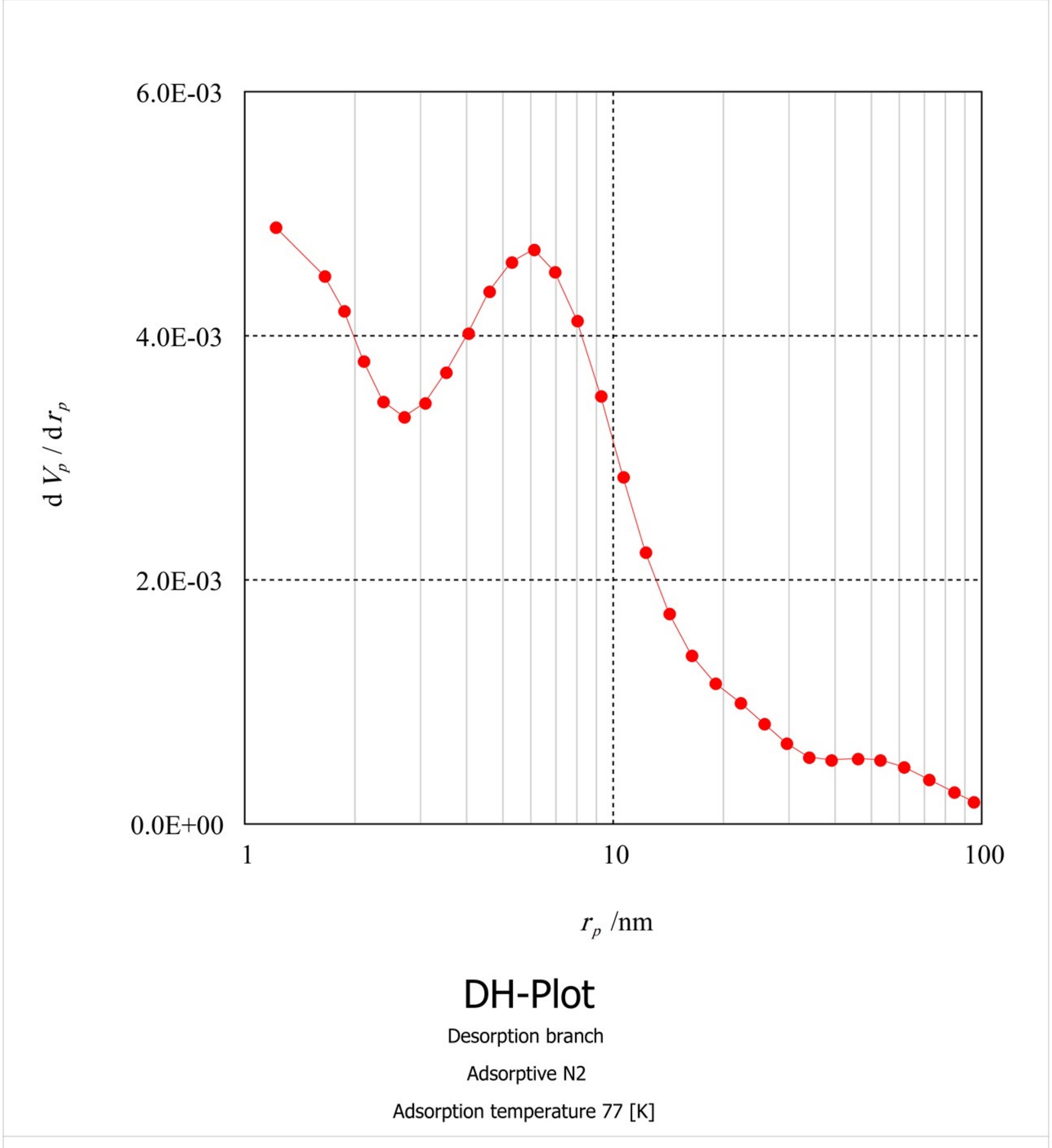

Figure S14 The DH plot for **2**.

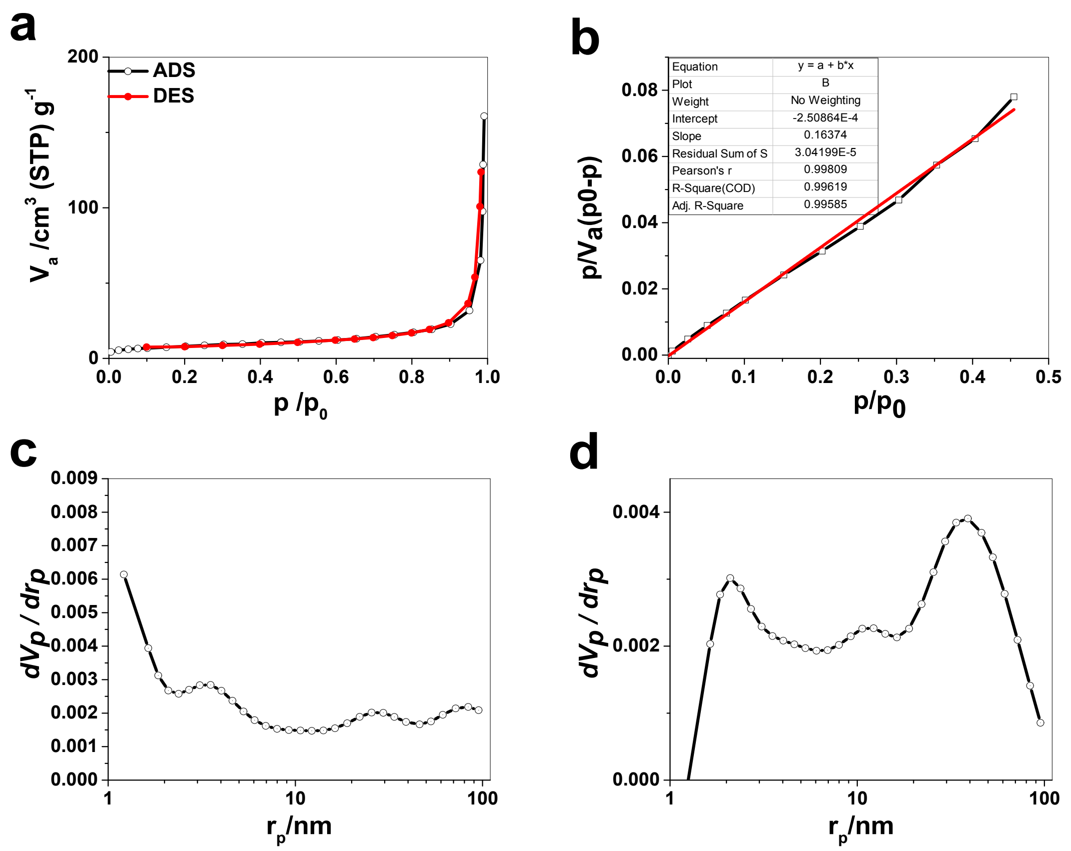

Figure 15 The nitrogen adsorption-desorption isotherms (a), Brunauer, Emmett, and Teller (BET) (b), Barrett, Joyner, and Halenda (BJH) (c) and DH (d) plots for **1**.

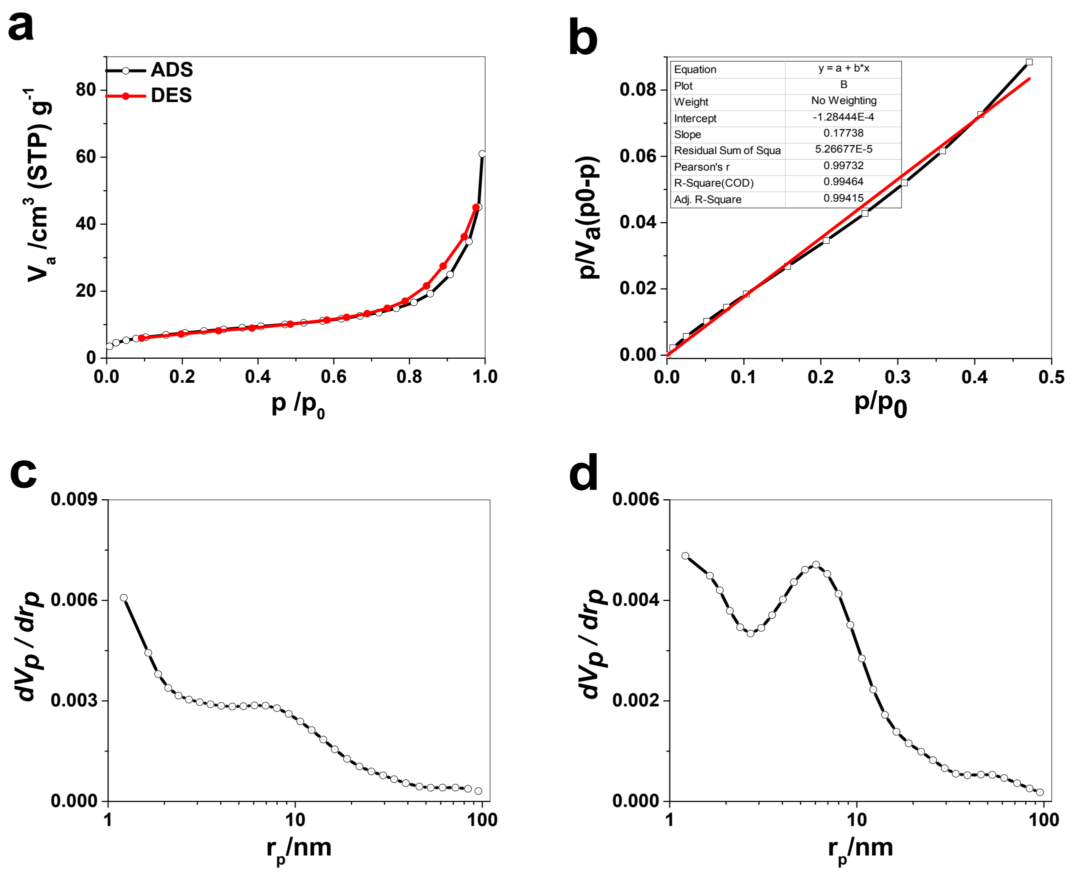

Figure 16 The nitrogen adsorption-desorption isotherms (a), Brunauer, Emmett, and Teller (BET) (b), Barrett, Joyner, and Halenda (BJH) (c) and DH (d) plots for **2**.
